# Venetoclax Combined with Azacitidine and Homoharringtonine in Relapsed/Refractory AML: A Multicenter, Phase 2 Trial

**DOI:** 10.1186/s13045-023-01437-1

**Published:** 2023-04-29

**Authors:** Hua Jin, Yu Zhang, Sijian Yu, Xin Du, Na Xu, Ruoyang Shao, Dongjun Lin, Yanqiu Chen, Jie Xiao, Zhiqiang Sun, Lan Deng, Xinquan Liang, Hongyu Zhang, Ziwen Guo, Min Dai, Pengcheng Shi, Fen Huang, Zhiping Fan, Zhao Yin, Li Xuan, Ren Lin, Xuejie Jiang, Guopan Yu, Qifa Liu

**Affiliations:** 1 Department of Hematology, Nanfang Hospital, Southern Medical University, Guangzhou, China; 2 Department of Hematology and Shenzhen Bone Marrow Transplantation Public Service Platform, Shenzhen Second People’s Hospital, The First Affiliated Hospital of Shenzhen University, Shenzhen, China; 3 Department of Hematology, The Seventh Affiliated Hospital of Sun Yat-Sen University, Shenzhen, China; 4 Department of Hematology, Maoming People’s Hospital, Maoming, China; 5 Department of Hematology, Sun Yat-Sen Memorial Hospital, Sun Yat-Sen University, Guangzhou, China; 6 Department of Hematology, Shenzhen Hospital, Southern Medical University, Shenzhen, China; 7 Department of Hematology, Shanghai Ninth People’s Hospital, Shanghai Jiao Tong University School of Medicine, Shanghai, China; 8 Department of Hematology, The First People’s Hospital of Chenzhou, Chenzhou, China; 9 Department of Hematology, Peking University Shenzhen Hospital, Shenzhen, China; 10 Department of Hematology, Zhongshan City People’s Hospital, Zhongshan, China; 11 Guangdong Provincial Key Laboratory of Digital Medicine and Biomechanics, Guangzhou, China

**Keywords:** Venetoclax, Azacitidine, Homoharringtonine, Relapsed, Refractory, Acute myeloid leukemia

## Abstract

**Background:**

Relapsed or refractory acute myeloid leukemia (R/R AML) has a dismal prognosis. The aim of this study was to investigate the activity and tolerability of venetoclax combined with azacitidine plus homoharringtonine (VAH) regimen for R/R AML.

**Methods:**

This phase 2 trial was done at ten hospitals in China. Eligible patients were R/R AML (aged 18–65 years) with an Eastern Cooperative Oncology Group performance status of 0–2. Patients received venetoclax (100 mg on day 1, 200 mg on day 2, and 400 mg on days 3–14) and azacitidine (75 mg/m^2^ on days 1–7) and homoharringtonine (1 mg/m^2^ on days 1–7). The primary endpoint was composite complete remission rate [CRc, complete response (CR) plus complete response with incomplete blood count recovery (CRi)] after 2 cycles of treatment. The secondary endpoints include safety and survival.

**Results:**

Between May 27, 2020, and June 16, 2021, we enrolled 96 patients with R/R AML, including 37 primary refractory AML and 59 relapsed AML (16 relapsed after chemotherapy and 43 after allo-HSCT). The CRc rate was 70.8% (95% CI 60.8–79.2). In the patients with CRc, measurable residual disease (MRD)-negative was attained in 58.8% of CRc patients. Accordingly, overall response rate (ORR, CRc plus partial remission (PR)) was 78.1% (95% CI 68.6–85.4). At a median follow-up of 14.7 months (95% CI 6.6–22.8) for all patients, median overall survival (OS) was 22.1 months (95% CI 12.7–Not estimated), and event-free survival (EFS) was 14.3 months (95% CI 7.0–Not estimated). The 1-year OS was 61.5% (95% CI 51.0–70.4), and EFS was 51.0% (95% CI 40.7–60.5). The most common grade 3–4 adverse events were febrile neutropenia (37.4%), sepsis (11.4%), and pneumonia (21.9%).

**Conclusions:**

VAH is a promising and well-tolerated regimen in R/R AML, with high CRc and encouraging survival. Further randomized studies are needed to be explored.

*Trial registration* clinicaltrials.gov identifier: NCT04424147.

**Supplementary Information:**

The online version contains supplementary material available at 10.1186/s13045-023-01437-1.

## Background

Acute myeloid leukemia (AML) is a heterogeneous and aggressive hematopoietic malignancy with a great variation in disease outcomes. Despite great advances in chemotherapy and hematopoietic stem cell transplantation (HSCT), there is still up to 35–45% of patients being refractory to treatments or relapsed [1]. The prognosis of refractory/relapsed (R/R) AML is dismal, with less than 10% overall survival (OS) at 3 years [2]. There is no standard salvage therapy for patients with R/R AML, indicating the urgent need for novel treatment to improve the outcomes [2–5]. Overexpression of the anti-apoptotic B-cell lymphoma 2 (BCL-2) family of proteins is a documented mechanism of resistance in AML and other malignancies [6, 7]. Venetoclax, an oral selective small-molecule BCL-2 inhibitor, in combination with hypomethylating agents (HMAs), such as azacitidine and decitabine, has been demonstrated to improve the outcomes in older or unfit patients with AML [8]. Resistance to venetoclax is mediated by other pro-survival proteins, such as myeloid-cell leukemia 1 (MCL1) and B-cell lymphoma-extra large (BCL-XL) [9]. HMAs might synergistically inhibit MCL1 and BCL-XL, thereby increasing the dependence of leukemia cells on BCL-2 [9]. However, retrospective and prospective studies showed that combinations of venetoclax and HMAs were less active in R/R AML treatment, with composite complete remission (CRc, complete response (CR) plus complete response with incomplete blood count recovery (CRi) rates of 11·6–46% [10–12].

Homoharringtonine, extracted from the herb *Cephalotaxus mannii* found in southern China, is an anti-leukemia drug and has been used in the treatment of AML and CML since 1970s [13–15]. It has been demonstrated that homoharringtonine reduces MCL1 expression, blocks short-half-life oncoproteins, and induces apoptosis in myeloid leukemia cells [16, 17]. In vitro and in vivo experiments showed that homoharringtonine had a synergistic anti-tumor effect with venetoclax through deeper inhibition of MCL1 in AML and diffuse large B-cell lymphoma [18, 19]. Our single small-sample exploratory study showed that venetoclax combined with azacitidine plus homoharringtonine (VAH) excelled venetoclax–azacitidine regimen in patients with R/R AML [20]. In vitro experiments, our data showed that VAH enhanced the anti-leukemia effect via deeper inhibition of MCL1, BCL-XL, and increased activation of BCL2 Associated X, Apoptosis Regulator (BAX) in AML cell lines (Additional file 1: Fig. S1). These data provide a strong clinical rationale for the VAH regimen for the treatment of R/R AML. Therefore, we set up a multicenter, phase 2 trial to investigate the efficacy and tolerability of VAH regimen for patients with R/R AML.

## Methods

### Study design and participants

In this multicenter, phase 2 trial, patients were enrolled at ten hospitals in China. Patients with R/R AML (aged 18–65 years) who had an Eastern Cooperative Oncology Group performance status of 0–2 were eligible for this trial. Refractory AML was defined as no CRc and a reduction in bone marrow (BM) blasts of less than 50% after one cycle or no CRc after two cycles. Relapsed AML was defined as recurrence of blasts in the peripheral blood (PB) or BM blasts ≥ 5% or development of extramedullary disease after achieving CRc [21]. Patients were excluded if they previously received venetoclax-based treatment, had acute promyelocytic leukemia, pregnancy, active acute or chronic graft-versus-host disease (GVHD), clinically significant coagulation abnormalities, clinically significant cardiovascular disease, uncontrolled active infection, substantial history of renal, neurological, pulmonary, psychiatric, endocrine, metabolic, immunological, hepatic, or any other medical conditions not suitable for the trial. Active acute GVHD or chronic GVHD was defined as GVHD requiring either at least 1 mg/kg per day of prednisone (or equivalent) or treatment beyond systemic corticosteroids [22]. The study protocol (Additional file 2: Appendix) was approved by the ethics committee review board at each of the ten hospitals, and written informed consent was obtained from patients or guardians in accordance with the Declaration of Helsinki before the initiation of the study.

### Procedures

VAH regimen consisted of 14-day courses of venetoclax and 7-day courses of azacitidine and homoharringtonine. Venetoclax began at 100 mg on day 1 and increased stepwise over 3 days to reach the target dose of 400 mg (100, 200, and 400 mg); dosing was continued at 400 mg per day from day 4 through day 14; azacitidine (75 mg/m^2^) and homoharringtonine (1 mg/m^2^) were administered subcutaneously on days 1 to 7. All patients were hospitalized during the treatments. Venetoclax dose was reduced by at least 50% in the patients receiving concomitant moderate or strong CYP3A4 inhibitors (e.g., azole antifungals) [23]. Fms-related receptor tyrosine kinase 3 (FLT3) inhibitors were recommended in the patients with FLT3 mutations before allo-HSCT. For the patients undergoing allo-HSCT, FLT3 inhibitors maintenance post-transplantation was recommended regardless of FLT3 being mutated or not. Once CRc was achieved, patients received allogeneic hematopoietic stem cell transplantation (allo-HSCT) if donors were available. If donors were unavailable, patients received one course of original therapy again and two or three cycles of cytarabine-based consolidation therapy. After consolidation, if patients have actionable target, they will receive targeted inhibitor maintenance such as FLT3 mutated patients receive sorafenib maintenance. If not, patients will receive pre-emptive treatment according to MRD detection. If patients did not reach CRc after two courses, they proceed to allo-HSCT if donors were available. If donors were unavailable, the patients might receive other salvage therapy. For patients who relapsed after allo-HSCT, donor lymphocyte infusion (DLI) was administered on day 15 after the initiation of the original therapy if donors were available, and the second DLI depended on GVHD status. The CD3^+^T cell count for each DLI was 3.0 × 10^7^/kg of the recipient weight. DLI was given monthly until GVHD occurred or MRD became negative or for a total of four times [24].

Criteria for removing patients from trial treatment included the development of intolerable adverse events related to study treatment, patient withdrew informed consent, and completion of the protocol therapy and evaluation period. The cytogenetic evaluation was used with standard metaphase karyotype and fluorescence in situ hybridization analysis. Molecular analysis via polymerase chain reaction and 167-gene institutional next-generation sequencing platform was performed at study enrollment. Measurable residual disease (MRD) was assessed by 8-color multiparameter flow cytometry (FC) using leukemia-associated immunophenotype or different from normal assessment with a minimum sensitivity of 10^–4^. The MRD levels of 0.01% were used as a threshold to distinguish MRD positive (MRD^**+**^) from MRD negative (MRD^−^) [25, 26].

For response assessments, BM was evaluated at cycle 1 day 28 and again 1–2 weeks after hematologic recovery if on day 28 BM was aplastic. Subsequent BM evaluations were done before and after cycle 2 and then, as clinically needed. Morphologic, cytogenetic, and MRD assessments were done during each BM assessment. Response criteria were defined by the European LeukemiaNet (ELN) 2017 guidelines. CR was defined as an absolute neutrophil count of more than 1000 cells per cubic millimeter, a platelet count of more than 100,000 per cubic millimeter, red-cell transfusion independence, and BM with less than 5% blasts. CRi was defined as all the criteria for CR, except for neutropenia (absolute neutrophil count, ≤ 1000 per cubic millimeter) or thrombocytopenia (platelet count, ≤ 100,000 per cubic millimeter) [21]. Partial remission (PR) was a minimal residual disease of 5% to 25% with a greater than 50% decrease in leukemic blast percentage. Non-remission (NR) was defined as a failure to obtain CRc or PR [21]. CRc comprised CR and CRi, and overall response rate (ORR) comprised CRc and PR.

Adverse events were defined as those that occurred from the first dose until 28 days after the discontinuation of treatment and were graded according to the National Cancer Institute Common Terminology Criteria for Adverse Events (CTCAE) version 4.0 [27]. An independent study adjudication committee (consisting of experts in hematology, infection, pathology, pharmacy, and statistics) judged whether adverse events were treatment-related or non-treatment-related.

### Outcomes

The primary endpoint was CRc after 2 cycles of trial treatment. The secondary endpoints were safety, overall survival (OS), event-free survival (EFS), disease-free survival (DFS), and relapse. OS was defined as the time from treatment until death or censored at the last follow-up. EFS was defined as the time from treatment until documented failure to achieve CRc, relapse after CRc, or death from any cause, whichever occurred first. DFS was defined as the time from the date of CRc until relapse, death from any cause, or censored at the last follow-up. Data for each patient were censored at the date of the last visit or the date on which the patient was last known to be alive.

### Statistical analysis

The sample size calculation for the trial was based on the assumption that the VAH regimen would achieve a higher CRc rate compared with historical CRc rate of 45% (on the basis of venetoclax in combination with HMAs study by our previous and others in R/R AML) [11, 20]. To identify a 15% absolute improvement in CRc with VAH regimen, a total of 87 patients were required to provide the study with a significance level of 5% and a power of 80%. After adjusting for a 10% dropout, the total planned sample size was 96 patients. The sample size calculation was done using PASS software, version 11·0.

The clinical data cutoff date was June 30, 2022. The descriptive analysis of patient characteristics included median and interquartile range (IQR) for continuous variables, and absolute and relative frequencies for categorical variables. The time-to-event endpoints including OS, EFS and DFS were analyzed by the Kaplan–Meier method and compared using the log-rank tests. The corresponding hazard ratio (HR) and 95% CI were estimated using the Cox proportional hazards model. The cumulative incidences of relapse were calculated by accounting for competing risks, and non-relapse mortality was a competing risk for relapse. The comparison of the cumulative incidence in the presence of a competing risk was done using the Fine and Gray model [28]. All variables in the Cox models were tested for proportional hazards assumption. Variables included in the univariable analysis were age, gender, AML status, ELN classification, MRD status, and allo-HSCT. Factors that were significant at the 0.1 level from the univariable model were included in the multivariable model. All statistics were analyzed in software R version 4.1.0 (R Development Core Team, Vienna, Austria) or Stata version 15.1 (StataCorp 4905 Lakeway Dr College Station, TX77845, USA) or SPSS version 22.0 (SPSS, Chicago, IL). All statistical tests were two-tailed with a significance level of 0.05. This trial is registered with ClinicalTrials.gov (NCT04424147).

## Results

### Patients and disposition

**Fig. 1:**
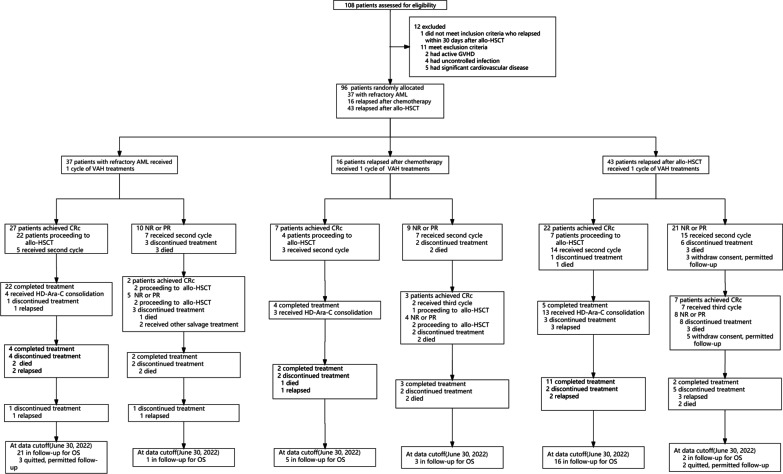
Trial profile. *HSCT* hematopoietic stem cell transplantation, *GVHD* graft-versus-host disease, *AML* acute myeloid leukemia, *VAH* Venetoclax Combined With Azacitidine And Homoharringtonine, *CRc* composite complete remission, *NR* non-remission, *PR* partial remission, *HD Ara-c* high-dose cytarabine, *OS* overall survival

Between May 27, 2020, and June 16, 2021, 108 patients with R/R AML were assessed for eligibility, 96 of whom were enrolled, including 37 (38.5%) patients with primary refractory AML and 59 (61.5%) with relapsed AML (16 (16.7%) relapsed after chemotherapy and 43 (44.8%) after allo-HSCT) (Fig. 1). There were 51 male (53.1%) and 45 female (46.9%), with a median age of 45 (IQR, 33–55) years at enrollment. Patient characteristics are shown in Table 1. Twenty-one (56.8%) patients with refractory AML had received one cycle, and 16 (43.2%) patients received two or more cycles of inducing therapy. Among patients with relapse after chemotherapy, 14 (87.5%) patients were first relapse, and 2 (12.5%) were second relapse. Among patients with relapse after allo-HSCT, 2 patients (4.65%) relapsed after a second allo-HSCT. Of the 96 patients enrolled, 45 patients (46.9%) received one cycle and 51 (53.1%) received more than one cycle, including 42 (82.4%) two and 9 (17.6%) three cycles as trail treatment. Among 19 patients with FLT3 mutations, 17 patients (89.5%) received sorafenib, and 2 (10.5%) gilteritinib treatment. More patients relapsed after allo-HSCT received more cycles of VEN-based treatments other than second allo-HSCT compared with primary refractory patients and patients relapsed after chemotherapy (*P* = 0.01 and *P* < 0.01).

**Table 1:** Baseline characteristics

Characteristics	All patients (*n* = 96)	Refractory AML (*n* = 37)	Relapsed AML after chemotherapy (*n* = 16)	Relapsed AML after allo-HSCT (*n* = 43)	*P*
Age, median (IQR), y	45 (33–55)	48 (38–57)	45.5 (33–64)	41 (32–52)	
Gender, No (%)					0.47
Male	51 (53.1)	21 (56.8)	10 (62.5)	20 (46.5)	
Female	45 (46.9)	16 (43.2)	6 (37.5)	23 (53.5)	
Prior hypomethylating agent^†^, No (%)					0.06
Yes	36 (37.5)	9 (24.3)	9 (56.3)	18 (41.9)	
No	60 (62.5)	28 (75.7)	7 (43.8)	25 (58.1)	
Cytogenetics^‡^, No (%)					0.55
Favorable	6 (6.3)	4 (10.8)	0 (0)	2 (4.7)	
Intermediate	53 (55.2)	18 (48.7)	10 (62.5)	25 (58.1)	
Poor	27 (28.1)	12 (32.4)	3 (18.8)	12 (27.9)	
Unknown	10 (10.4)	3 (8.1)	3 (18.8)	4 (9.3)	
ELN classification, No (%)					0.68
Favorable	17 (17.7)	7 (18.9)	2 (12.5)	8 (18.6)	
Intermediate	22 (22.9)	10 (27.0)	5 (31.3)	7 (16.3)	
Adverse	57 (59.4)	20 (54.1)	9 (56.3)	28 (65.1)	
Number of VEN cycles, No (%)					0.01
One	45 (46.9)	25 (67.6)	6 (37.5)	14 (32.6)	
Two	42 (43.7)	12 (32.4)	8 (50.0)	22 (51.2)	
Three	9 (9.4)	0 (0)	2 (12.5)	7 (16.3)	
Patients bridge to Allo-HSCT, No (%)	40 (41.7)	26 (70.3)	7 (43.8)	7 (16.3)	< 0.01
Molecular abnormalities, No (%)					
NPM1	11 (11.5)	3 (8.1)	2 (12.5)	6 (14.0)	0.71
AML1-ETO	6 (6.3)	4 (10.8)	0 (0)	2 (4.7)	0.28
CEBPA	10 (10.4)	3 (8.1)	2 (12.5)	5 (11.6)	0.84
TET2	41 (42.7)	13 (35.1)	7 (43.8)	21 (48.8)	0.46
DNMT3A	18 (18.8)	9 (24.3)	2 (12.5)	7 (16.3)	0.51
IDH1/2	14 (14.6)	8 (21.6)	3 (18.8)	3 (7.0)	0.16
FLT3	19 (19.8)	7 (18.9)	2 (12.5)	10 (23.3)	0.64
ASXL1	22 (22.9)	7 (18.9)	4 (25.0)	11 (25.6)	0.76
RUNX1	22 (22.9)	6 (16.2)	3 (18.8)	13 (30.2)	0.30
TP53	7 (7.3)	4 (10.8)	0 (0)	3 (7.0)	0.38
MLL	7 (7.3)	2 (5.4)	3 (18.8)	2 (4.7)	0.15
EZH2	6 (6.3)	1 (2.7)	1 (6.3)	4 (9.3)	0.48
BCL6	7 (7.3)	1 (2.7)	1 (6.3)	5 (11.6)	0.31
BCOR	7 (7.3)	6 (16.2)	0 (0)	1 (2.3)	0.03
GATA2	5 (5.2)	1 (2.7)	0 (0)	4 (9.3)	0.25
RAS	9 (9.4)	6 (16.2)	1 (6.3)	2 (4.7)	0.19
CD101	8 (8.3)	2 (5.4)	1 (6.3)	5 (11.6)	0.57

Data are number of patients (%) or median (IQR)

*AML* acute myeloid leukemia, *Allo-HSCT* allogeneic hematopoietic stem cell transplantation, *ELN* European Leukemia Net, *VEN* venetoclax

^†^Prior hypomethylating agent included azacitidine in 21 patients and decitabine in 15 patients

^‡^Cytogenetic risks were based on 2017 European Leukemia Net risk stratification

### Efficacy

**Fig. 2:**
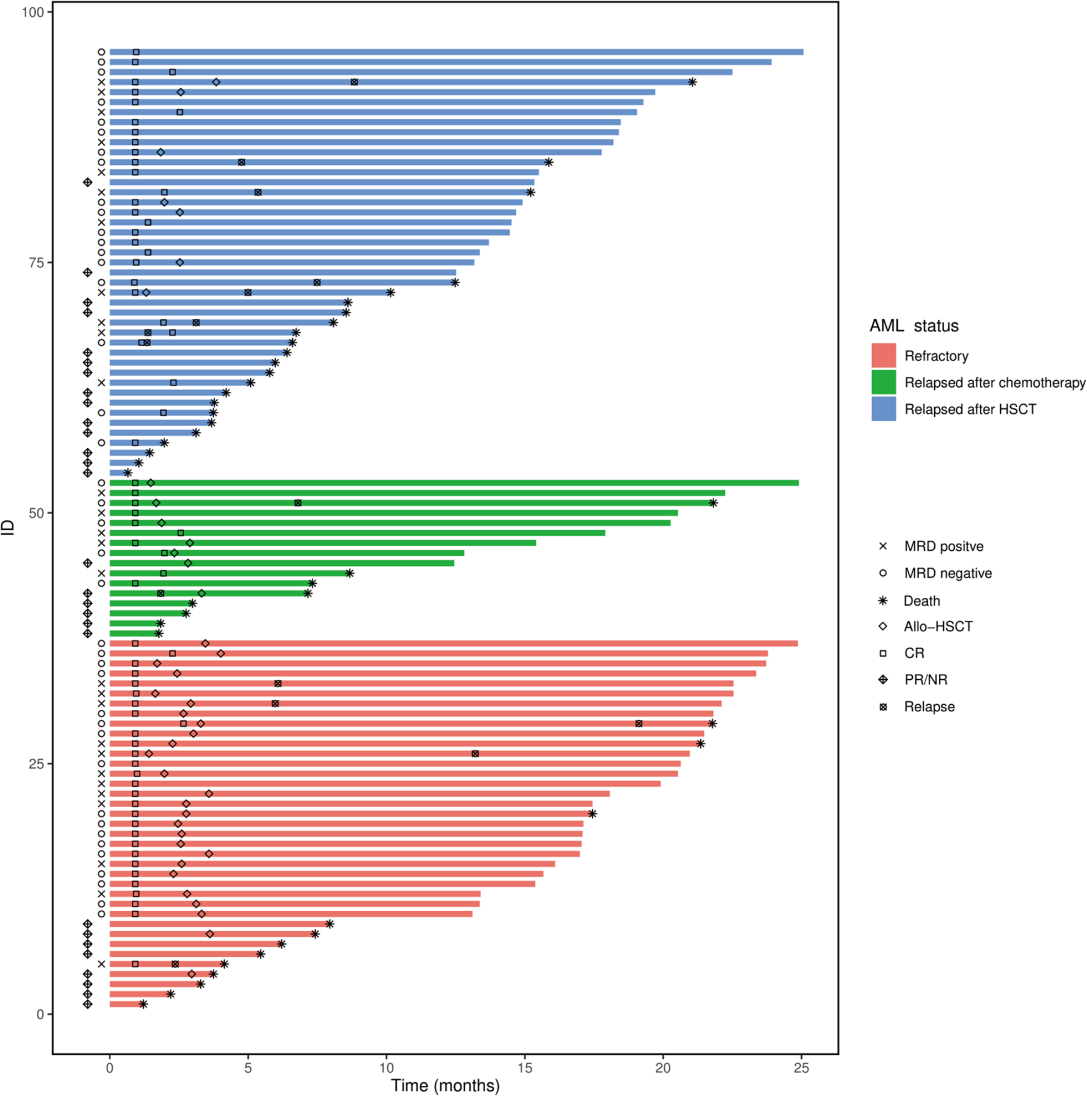
Swimmer plot of dynamic response assessment. Each bar is an individual patient. *AML* acute myeloid leukemia, *HSCT* hematopoietic stem cell transplantation, *MRD* measurable residual disease, *CR* complete remission, *NR* non-remission, *PR* partial remission

The responses of treatments are summarized in Table 2 and Fig. 2. CRc rate at the end of cycle 2 was 70.8% (68 of 96 patients; 95% CI 60.8–79.2), with CRc at the end of cycle 1 was 58.3% (56 of 96 patients; 95% CI 48.1–67.9). In the patients with CRc, MRD^−^ was attained in 58.8% of CRc patients. Accordingly, ORR at the end of cycle 2 was 78.1% (75 of 96 patients; 95% CI 68.6–85.4), with ORR at the end of cycle 1 was 71.9% (69 of 96 patients; 95%CI 61.9–80.1). There were no difference in CRc and ORR among patients with or without prior HMAs exposure (CRc, 63.9% (95%CI 46.8–78.1%) vs 75.0% (62.3–84.5%), *p* = 0.246; ORR, 75.0% (95%CI 58.0–86.7%) vs 80.0% (67.7–88.4%), *p* = 0.566). Of the 68 patients reached CRc, 36 patients (52.9%) proceeded to allo-HSCT, 31 (45.6%) received a median of 3 courses (range 1–4) of consolidation chemotherapy, and 1 patient (1.5%) gave up further consolidation treatment due to personal reason. Of the 28 patients who did not obtain CRc, 20 patients (71.4%) received other salvage treatments (16 chemotherapy and 4 allo-HSCT), and 8 (28.6%) gave up treatments or died. Of the 20 patients receiving other salvage treatments, 6 patients (30.0%) obtained CRc (4 allo-HSCT and 2 chemotherapy). Among the 40 patients bridged to allo-HSCT, 36 (90.0%) received sorafenib maintenance post-transplantation, except 4 patients (10.0%) (2 patients GVHD and 2 patients haematotoxicity). Of the 43 patients relapsed after allo-HSCT, 34 patients (79.1%) received a median of two DLI (range 1–2). Twenty-six patients (76.5%) developed the acute GVHD, and six patients (17.6%) developed the extensive chronic GVHD among 34 patients who received DLI treatment.

**Table 2:** Response outcomes

	All(*n* = 96)	Refractory AML (*n* = 37)	Relapsed AML after chemotherapy (*n* = 16)	Relapsed AML after allo-HSCT (*n* = 43)	*P*
CRc (CR + CRi), No. (% [95% CI])	68 (70.8[60.8–79.2])	29 (78.4[61.9–89.0])	10 (62.5[36.6–82.8])	29 (67.4[51.9–79.9])	0.407
CR, No. (%)	40 (41.7)	19 (51.4)	8 (50.0)	13 (30.2)	
CRi, No. (%)	28 (29.2)	10 (27.0)	2 (12.5)	16 (37.2)	
MRD- CRc, No. (%)^†^	40 (58.8)	17 (58.6)	5 (50.0)	18 (62.1)	0.799
PR, No. (%)	7 (7.3)	1 (2.7)	1 (6.3)	5 (11.6)	
NR, No. (%)	21 (21.9)	7 (18.9)	5 (31.3)	9 (20.9)	
ORR, No. (% [95% CI])	75 (78.1[68.6–85.4])	30 (81.1[64.8–90.9])	11 (68.8[42.1–86.9])	34 (79.1[64.0–88.9])	0.596
CRc at Cycle 1, No. (% [95% CI])	56 (58.3[48.1–67.9])	27 (73.0[56.2–85.0])	7 (43.8[21.7–68.6])	22 (51.2[36.2–65.9])	0.062
ORR at Cycle 1, No. (% [95% CI])	69 (71.9[61.9–80.1])	30 (81.1[64.8–90.9])	9 (56.3[31.4–78.3])	30 (69.8[54.2–81.8])	0.167
EFS					
Median, months (95% CI)	14.3 (7.0 to NE)	Not reached	7.8 (2.0 to NE)	6.0 (2.3 to NE)	0.182
12-months, % (95% CI)	51.0 (40.7–60.5)	64.9 (47.3–77.9)	43.8 (19.8–65.6)	41.9 (27.1–55.9)	0.099
Estimated 24-months, % (95% CI)	46.0 (34.0–57.2)	54.1 (32.8–71.2)	43.8 (19.8–65.6)	41.9 (27.1–55.9)	0.182
OS					
Median, months (95% CI)	22.1 (12.7 to NE)	Not reached	22.1 (3.0 to NE)	15.4 (6.8 to NE)	0.114
12-months, % (95% CI)	61.5 (51.0–70.4)	70.3 (52.8–82.3)	56.3 (29.5–76.2)	55.8 (39.9–69.1)	0.345
Estimated 24-months, % (95% CI)	47.2 (33.3–59.8)	63.2 (41.9–78.6)	37.5 (8.4–67.8)	34.3 (14.6–56.4)	0.114

Data are number of patients (%)

*AML* acute myeloid leukemia, *Allo-HSCT* allogeneic hematopoietic stem cell transplantation, *CRc* composite complete remission, *CR* complete remission, *CRi* CR with incomplete hematological recovery, *MRD* minimal residual disease, *PR* partial remission, *NR* non-remission, *ORR* overall response rate, *EFS* event-free survival, *OS* overall survival

^†^MRD measured in patients who achieved CRc using multicolour flow cytometry validated to a sensitivity level of 0·01%. NE, not estimated

### Cytogenetic and molecular response

**Fig. 3:**
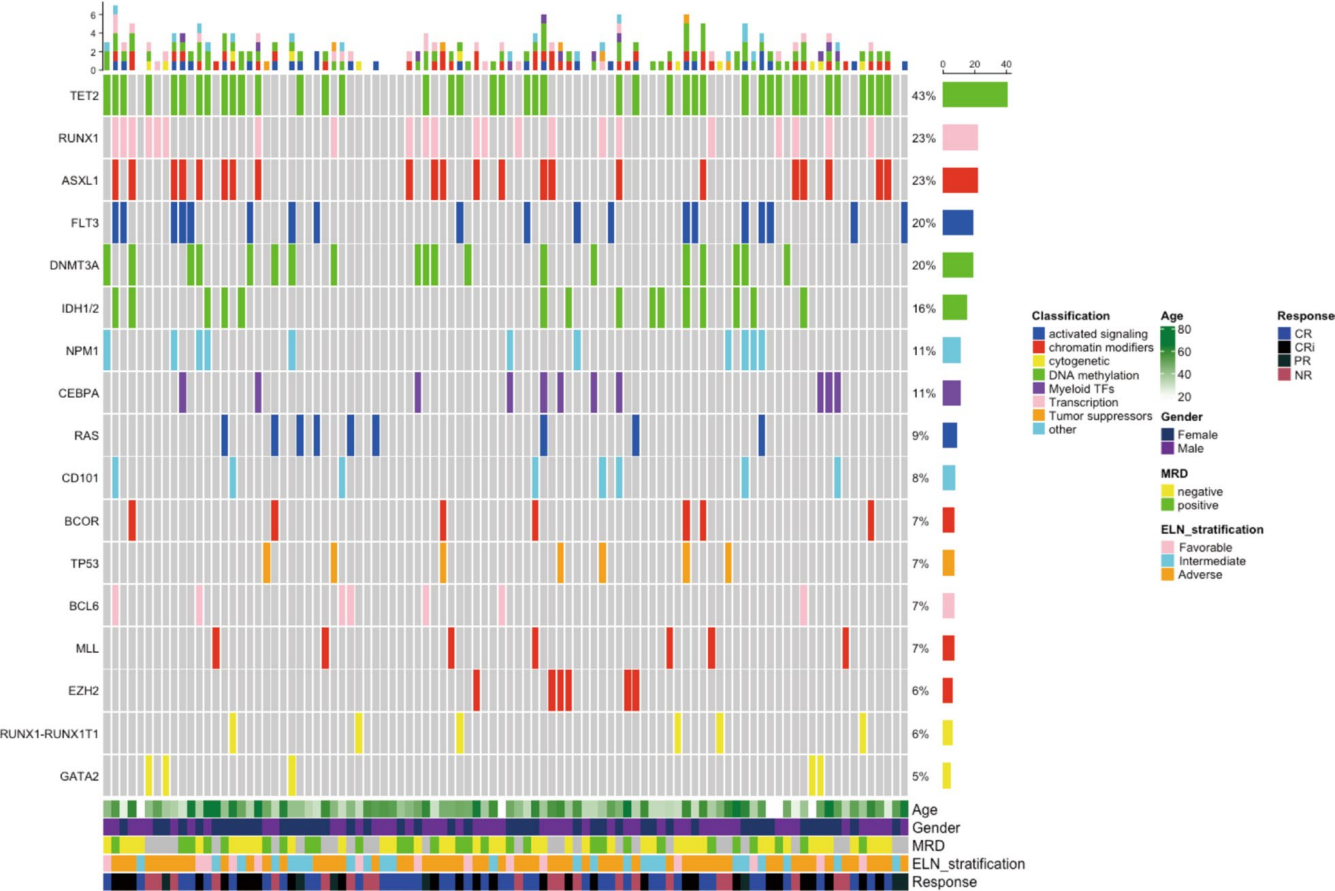
Mutational landscapes of 96 patients with refractory /relapsed AML. *CR* complete remission, *CRi* CR with incomplete hematological recovery, *PR* partial remission, *NR* non-remission, *MRD* measurable residual disease, *TFs* transcription factors

The results of cytogenetic and molecular subgroup analyses with respect to CRc are shown in Additional file 1: Table S1 and Fig. 3. CRc was 94.1% (95%CI 66.0–99.2), 63.6% (41.6–81.2) and 66.7% (53.3–77.8) in the ELN favorable-, intermediate- and adverse-risk groups, respectively (*P* = 0.064). The CRc in favorable-risk group was significantly better than intermediate- or adverse-risk groups (*P* = 0.025; *P* = 0.025, respectively). In the cytogenetic subgroups, CRc was 100%, 77.4% (63.9–86.8) and 55.6% (36.3–73.2) in the favorable-, intermediate- and poor-risk groups, respectively (*P* = 0.066). The CRc in poor-risk group was interior than favorable- or intermediate-risk groups (*P* = 0.044; *P* = 0.041, respectively). We further analyzed the molecular mutation subgroups which mutation rate was more than 5% according to related literature report and our samples size [29]. Nine patients (9.4%) harbored K/NRAS^mut^ and 7 patients (7.3%) harbored MLL^mut^. The CRc was 44.4% (16.3–76.7) for K/NRAS^mut^ and 42.9% (12.7–79.4) for MLL^mut^. Except for K/NRAS^mut^ and MLL^mut^, the CRc were above 50% in all the other molecular mutation. The CRc was not significantly different between patients with and without these mutations, except for K/NRAS^mut^ and MLL^mut^ (Additional file 1: Fig. S2).

### Survival

**Fig. 4:**
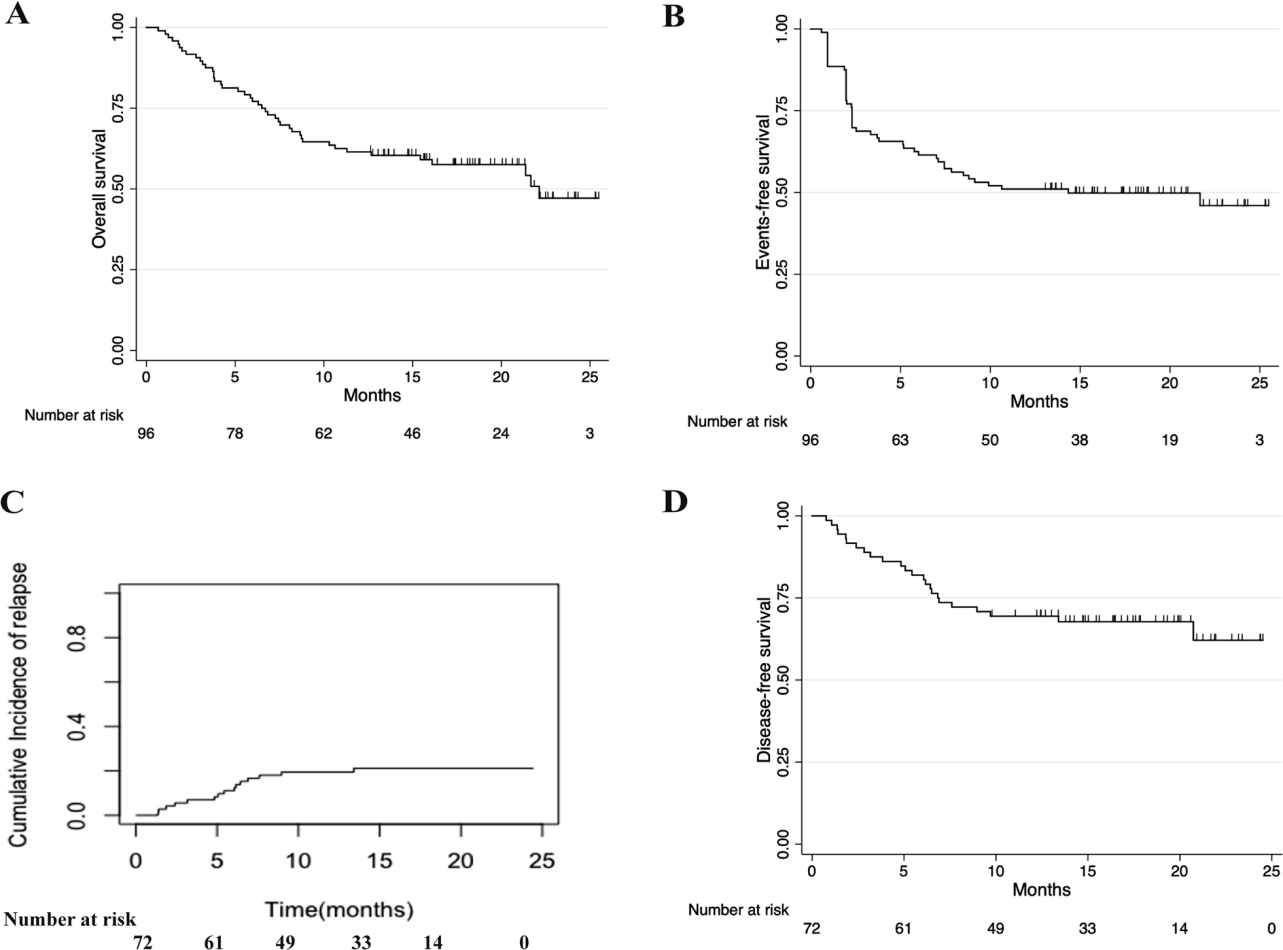
Cumulative incidence of overall survival (**A**), event-free survival (**B**), relapse(**C**) and disease-free survival (**D**)

**Fig. 5:**
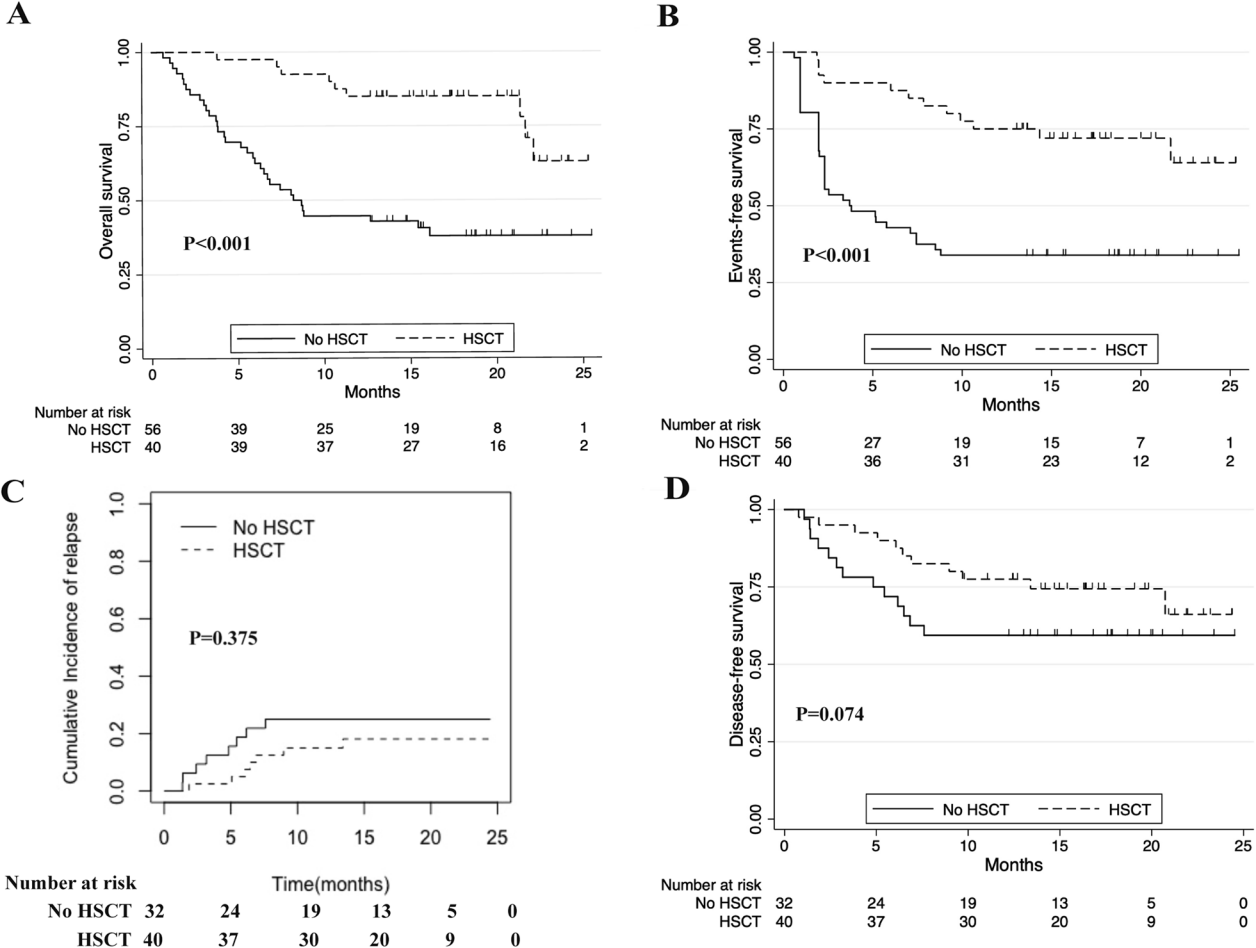
Cumulative incidence of overall survival (**A**), event-free survival (**B**), relapse(**C**) and disease-free survival (**D**) among patients who received or did not receive allo-HSCT. HSCT, hematopoietic stem cell transplantation

Within a median follow-up of 14.7 months (IQR, 6.6–22.8), 43 patients (44.8%) died. The most common causes of death were relapse/disease progression (*n* = 36, 83.7%), infections (sepsis [*n* = 2], pneumonia [*n* = 1], febrile neutropenia [*n* = 1]), GVHD (*n* = 2), and heart failure (*n* = 1). The median OS was 22.1 months (95% CI 12.7–Not estimated (NE)), including the median OS was not reached (95% CI 22.1–NE) for patients achieving CRc and 3.8 months (95% CI 3.0–6.3) for patients who did not achieve CRc. The median EFS was 14.3 months (95% CI 7.0–NE). The 1-year OS was 61.5% (95% CI 51.0–70.4), and 1-year EFS was 51.0% (95% CI 40.7–60.5) (Table 2 and Fig. 4A, B). With the median time of 5.4 months (IQR 2.4–6.9), 14 patients (20.6%) relapsed among the 68 patients who achieved CRc. The 1-year cumulative incidence of relapse was 19.4% (95% CI 11.2–29.4). Accordingly, 1-year DFS was 69.4% (95% CI 57.4–78.7) (Fig. 4C, D). The OS, EFS, relapse and DFS of disease subgroup are shown in Additional file 1: Fig. S3. Of the 96 patients, 40 patients (41.7%) bridged to allo-HSCT, including 36 patients (90.0%) achieved CR and 4 patients (10.0%) did not achieve CR. The 1-year OS was 85.0% (95% CI 69.6–93.0) versus 44.6% (95% CI 31.4–57.0) for patients who received or did not receive allo-HSCT (*P* < 0.001, Fig. 5A) The 1-year EFS was 75.0% (95% CI 58.5–85.7) versus 33.9% (95% CI 22.0–46.3) (*P* < 0.001). The 1-year cumulative incidence of relapse was 15.0% (95% CI 6.0–27.8) and 25.0% (95% CI 11.6–41.0) for patients who received or did not receive allo-HSCT (*P* = 0.375). Accordingly, 1-year DFS was 77.5% (95% CI 61.2–87.6) versus 59.4% (95% CI 40.5–74.0) (*P* = 0.074) (Fig. 5B–D). Among patients who reached CRc, the 1-year OS was 91.7% (95% CI 76.4–97.2) and 71.9% (95% CI 52.9–84.3; *P* = 0.023) for patients who received or did not receive allo-HSCT. Accordingly, the 1-year DFS was 83.3% (95% CI 66.6–92.1) and 59.4% (95% CI 40.5–74.0), respectively (*P* = 0.016) (Additional file 1: Fig. S4A, B). Exploratory subgroup analyses showed that 1-year OS was 58.4% (95% CI 46.6–68.5) for patients without FLT3^mut^ and 73.7% (95% CI 47.9–88.1) for patients with FLT3^mut^ (*P* = 0.266). The 1-year EFS was 51.9% (95% CI 40.3–62.4) and 47.4% (95% CI 24.4–67.3), respectively (*P* = 0.823). The 1-year cumulative incidence of relapse was 16.4% (95% CI 8.0–27.4) for patients without FLT3^mut^ and 29.4% (95% CI 10.1–52.0) for patients with FLT3^mut^ (*P* = 0.248). Accordingly, 1-year DFS was 72.7% (95% CI 58.9–82.6) and 58.8% (95% CI 32.5–77.8), respectively (*P* = 0.258). (Additional file 1: Fig. 5A–D). A post hoc multivariable analysis showed that proceeding to allo-HSCT and MRD^−^ were protective factors for OS (HR 0.36 (95%CI 0.13–0.98); *P* = 0.046, and HR 0.35 (95%CI 0.13–0.93); *P* = 0.035. Additional file 1: Table S2).

### Adverse events

Common adverse events are summarized in Table 3. The most frequently reported hematologic adverse events of grade 3 or higher included neutropenia (82.3%), thrombocytopenia (75.0%), anemia (66.7%), and febrile neutropenia (37.4%). Gastrointestinal adverse events of any grade were common and predominantly included nausea (26.0%), constipation (12.5%), diarrhea (11.5%), and vomiting (12.5%). Notable serious adverse events (grade ≥ 3) were febrile neutropenia (37.4%), sepsis (11.4%), pneumonia (21.9%), and heart failure (4.1%). Tumor lysis syndrome was reported during the ramp-up period (on days 1 through 3 when the dose of venetoclax was increased) in 2 patients (2.0%).

**Table 3:** Treatment-related adverse events

	Grade 1–2	Grade 3	Grade 4	Grade 5
*Adverse event* (*n* = 96)				
Anemia	16 (16.7)	62 (64.6)	2 (2.1)	0
Neutropenia	6 (6.3)	60 (62.5)	19 (19.8)	0
Thrombocytopenia	8 (8.3)	14 (14.6)	58 (60.4)	0
Febrile neutropenia	0	25 (26.0)	10 (10.4)	1 (1.0)
Pneumonia	1 (1.0)	15 (15.6)	6 (6.3)	1 (1.0)
Sepsis	0	0	10 (10.4)	1 (1.0)
Nausea	20 (22.9)	3 (3.1)	0	0
Constipation	12 (12.5)	0	0	0
Diarrhea	11 (11.5)	0	0	0
Vomiting	10 (10.4)	2 (2.1)	0	0
Decreased appetite	22 (22.9)	2 (2.1)	0	0
Hypokalemia	13 (13.5)	5 (5.2)	1 (1.0)	0
Peripheral edema	10 (10.4)	1 (1.0)	0	0
Fatigue	23 (24.0)	2 (2.1)	0	0
Mucositis	8 (8.3)	4 (4.2)	0	0
Colitis	5 (5.2)	1 (1.0)	0	0
Cough	11 (11.5)	1 (1.0)	0	0
Muscle weakness	0	1 (1.0)	0	0
Hyperbilirubinemia	9 (9.4)	3 (3.1)	0	0
ALT or AST elevation	12 (12.5)	4 (4.2)	0	0
Allergic reaction	3 (3.7)	1 (1.0)	0	0
Heart failure	1 (1.0)	2 (2.1)	1 (1.0)	1 (1.0)
Renal failure	0	0	1 (1.0)	0
Tumor lysis syndrome	0	1 (1.0)	1 (1.0)	0

All patients in the safety population included (*n* = 96). Toxicity grades are according to the Common Terminology Criteria for Adverse Events version 4.03. Listed toxicities include both those attributable to therapy and those deemed not attributable to therapy. Data are *n* (%)

The percentage of patients who discontinued VAH owing to adverse events was 9.4%, including sepsis (*n* = 3), pneumonia (*n* = 3), heart failure (*n* = 2), and bleeding (*n* = 1). The delay between cycles and dose reduction owing to adverse events occurred in 29.2% of the patients. The delay between cycles and dose reduction was primarily because of myelosuppression, including neutropenia (5.2%), febrile neutropenia (19.8%), and thrombocytopenia (4.2%). Treatment-related death was 4.2%, including sepsis (*n* = 1), pneumonia (*n* = 1), febrile neutropenia (*n* = 1), and heart failure (*n* = 1).

## Discussion

This multicenter, phase 2 single-arm study provides the first evidence that VAH regimen has a robust CRc and encouraging OS in patients with R/R AML. Furthermore, VAH regimen is a well-tolerated regimen.

Reported CRc rates of venetoclax-based treatments for R/R AML varied greatly, ranging from 20 to 67% [12, 30–35]. Treatment responses were associated with many factors, such as the patients’ baseline characteristics, genetic characteristics, and venetoclax-based regimens and so on. It was reported that CRc of venetoclax-based two-agent regimen was 38.5–46.0% for R/R AML patients [11, 29, 35]. Kantarjian et al. reported CRc rate was 61% in venetoclax combined with FLAG-IDA regimen treatment (fludarabine, cytarabine, G-CSF, and idarubicin) [31]. The CRc rate of venetoclax and cytarabine with idarubicin was 67% as salvage therapy for children with R/R AML [32]. Homoharringtonine-based salvage regimens for R/R AML were recommended by Chinese 2021 treatment guidelines [36]. An early exploratory study showed that the remission rate of homoharringtonine combined with cytarabine regimen was 22.7% for R/R AML patients [14], and a meta-analysis revealed that the CR rate of homoharringtonine combined with cytarabine plus granulocyte colony-stimulating factor (G-CSF) was around 50% [15]. A case report demonstrated that two patients with R/R AML achieved CR with dose-adjusted homoharringtonine, cytarabine and G-CSF combined with venetoclax–azacitidine regimen [37]. In this study, VAH regimen showed high CRc of 70.8% for R/R AML patients. It might be superior to venetoclax–azacitidine regimen or homoharringtonine-based regimen [11, 14, 15, 29, 35]. Although cross-trial comparisons might be made with caution, the CRc of patients receiving VAH was compared favorably when taken in the context of published studies of venetoclax combined with intensive chemotherapy in R/R AML patients, in which CRc rate was 61–67% [31, 32]. It was reported that patients with FLT3-mutated R/R AML had inferior response to venetoclax therapy [32, 34]. Recently, Naval et al. reported CRc was 40% with venetoclax plus gilteritinib treatment [38] and Maiti et al. reported CRc was 63% with venetoclax–azacitidine plus FLT3 inhibitor treatment [39]. In this study, all 19 patients with FLT3 mutations received FLT3 inhibitors, with the CRc of 78.9%. In addition to achieving a high CRc rate, we further observed 58.8% of patients attained MRD negative. This deeper remission might further translate into survival advantage, with evidence that MRD^−^ status was a protective factor for OS in multivariate analysis.

The collaboration mechanism of homoharringtonine and venetoclax in anti-leukemia effect has been recently demonstrated [19]. Xie et al. reported that homoharringtonine combined with venetoclax downregulated MCL-1 by inhibiting p-ERK and activating BAX [19]. In accordance with the previous study, our study also showed that homoharringtonine synergized with venetoclax to deeply inhibit MCL1 and BCL-XL, and VAH increased the activation of BAX in AML cell lines (Additional file 1: Fig. S1). Allo-HSCT is a cure modality for R/R AML patients [40–42]. In this study, 40 patients who bridged to allo-HSCT achieved the 1-year OS of 85.0%, which compared favorably with 44.6% in patients who did not bridge to allo-HSCT. Our results suggested that VAH regimen followed by allo-HSCT might be effective to realize long-term survival for R/R AML patients.

Some studies including our own report suggested that the sensitivity to venetoclax-based therapy was related with molecular mutations of AML [11, 32, 34, 43, 44]. AML patients with IDH1/2^mut^, NPM1^mut^, RUNX1^mut^, TET2^mut^, ASXL1^mut^, or SRSF2^mut^ responded well to the venetoclax-based therapy, while those with FLT3^mut^, TP53^mut^, K/NRAS^mut^, SF3B1^mut^ or DNMT3A^mut^ experienced poor response [11, 34, 43]. In this study, our results align with the previous reports that patients with K/NRAS^mut^ had inferior response, and patients with IDH1/2^mut^, NPM1^mut^, RUNX1^mut^, TET2^mut^ and ASXL1^mut^ responded well [11, 34, 43]. Whereas, inconsistent with those reports, patients with FLT3^mut^ or DNMT3A^mut^ showed favorable response, suggesting that VAH regimen might overcome the poor prognosis of FLT3^mut^ or DNMT3A^mut^ [11, 34, 43]. For the patients with FLT3^mut^, the high CRc might be attributed to the synergistic anti-tumor effect of VAH combined with FLT3 inhibitors [19, 45–48]. It has been demonstrated that BCL-2 inhibitor combined with homoharringtonine markedly inhibits the expression of p-FLT3 and its downstream signaling proteins, p-Stat5 and MCL-1, inducing apoptosis in AML cell lines [19, 49]. Zhang et al. reported that FLT3-ITD mutated patients might benefit from the homoharringtonine plus sorafenib therapy clinically [45]. In vitro studies showed that FLT3 inhibitor had a synergistic anti-tumor effect with venetoclax [46–48, 50]. Therefore, to further confirm the efficacy, our new trial of VAH plus FLT3 inhibitor for FLT3^mut^ R/R AML is ongoing. The potential mechanism of VAH regimen’s favorable response in DMNT3A^mut^ AML might be due to that homoharringtonine inhibited mTOR activation pathway which initiated by DNMT3A mutation [51].

One of the main concerns when combining triplet agents was the potential for increased side-effect profile, especially myelosuppression and infections. The venetoclax was administered on D1-28 in the venetoclax-based two-drug combination regimen for R/R AML [8, 29]. Venetoclax was given for 7–14 days per course in the venetoclax-based three drug or four drug combination regimen for high-risk myelodysplastic syndrome or R/R AML [31, 52]. To acknowledge that, we implemented a reduced venetoclax dosing from 28 to 14 days per course. The goal was to maximize the potentiation of venetoclax during the period of combination chemotherapy and allow sufficient time for marrow recovery. The results of VAH regimen were encouraging. The VAH regimen was well tolerated with low treatment-related mortality. The times to count recovery and infections were similar to other venetoclax-based treatment [29, 31, 32]. Grade 3–4 adverse events were mainly febrile neutropenia and infectious complications. The grade 4–5 febrile neutropenia and treatment-related death was 11.4% and 4.2%, respectively. Although cross-trial comparisons might be made with caution, the febrile neutropenia and treatment-related death rates were similar to other venetoclax-based combination therapy for AML [29, 52].

Our study has some limitations. It is a single-arm trial that limit the conclusions. Although our median follow-up is more than a year, longer follow-up is needed to confirm the durability of the responses and long-term survival.

## Conclusions

In summary, this study represents the first study to explore the efficacy and safety of VAH regimen in R/R AML. VAH regimen is a promising, and well-tolerated regimen in R/R AML, with high CRc rates and encouraging survival. This study can provide the basis for future randomized comparisons to help confirm the benefit.

## Supplementary Information

**Additional file 1.** In vitro experiments and subgroup analaysis.

**Additional file 2.** Clinical study protocol.

## Data Availability

The datasets used and/or analyzed during the current study are available from the corresponding author on reasonable request.

## Abbreviations

AML: Acute myeloid leukemia
HSCT: Hematopoietic stem cell transplantation
R/R: Refractory/relapsed
OS: Overall survival
BCL-2: B-cell lymphoma 2
HMAs: Hypomethylating agents
MCL1: Myeloid-cell leukemia 1
BCL-XL: B-cell lymphoma-extra large
CRc: Composite complete remission
VAH: Venetoclax combined with azacitidine plus homoharringtonine
venetoclax–azacitidine: Venetoclax combined with azacitidine
BAX: BCL2 associated X, apoptosis regulator
BM: Bone marrow
GVHD: Graft-versus-host disease
FLT3: Fms-related receptor tyrosine kinase 3
allo-HSCT: Allogeneic hematopoietic stem cell transplantation
DLI: Donor lymphocyte infusion
MRD: Measurable residual disease
FC: Flow cytometry
CR: Complete remission
CRi: CR with incomplete hematological recovery
PR: Partial remission
NR: Non-remission
ORR: Overall response rate
CTCAE: Common Terminology Criteria for Adverse Events
EFS: Event-free survival
DFS: Disease-free survival
IQR: Interquartile range
HR: Hazard ratio
ELN: European leukemia net
FLAG-IDA: Fludarabine, cytarabine, G-CSF, and idarubicin
HA: Homoharringtonine combined with cytarabine
HAG: Homoharringtonine, cytarabine and granulocyte colony-stimulating factor
G-CSF: Granulocyte colony-stimulating factor
