# Supplementary material for: Venetoclax Combined with Azacitidine and Homoharringtonine in Relapsed/Refractory AML: A Multicenter, Phase 2 Trial
Source: J Hematol Oncol. 2023 Apr 29;16:42. doi: 10.1186/s13045-023-01437-1 (PMC10149010; doi:10.1186/s13045-023-01437-1)
Supplement: Supplementary file 1 — Additional file 1. In vitro experiments and subgroup analaysis. [file 13045_2023_1437_MOESM1_ESM.docx]

**S1**
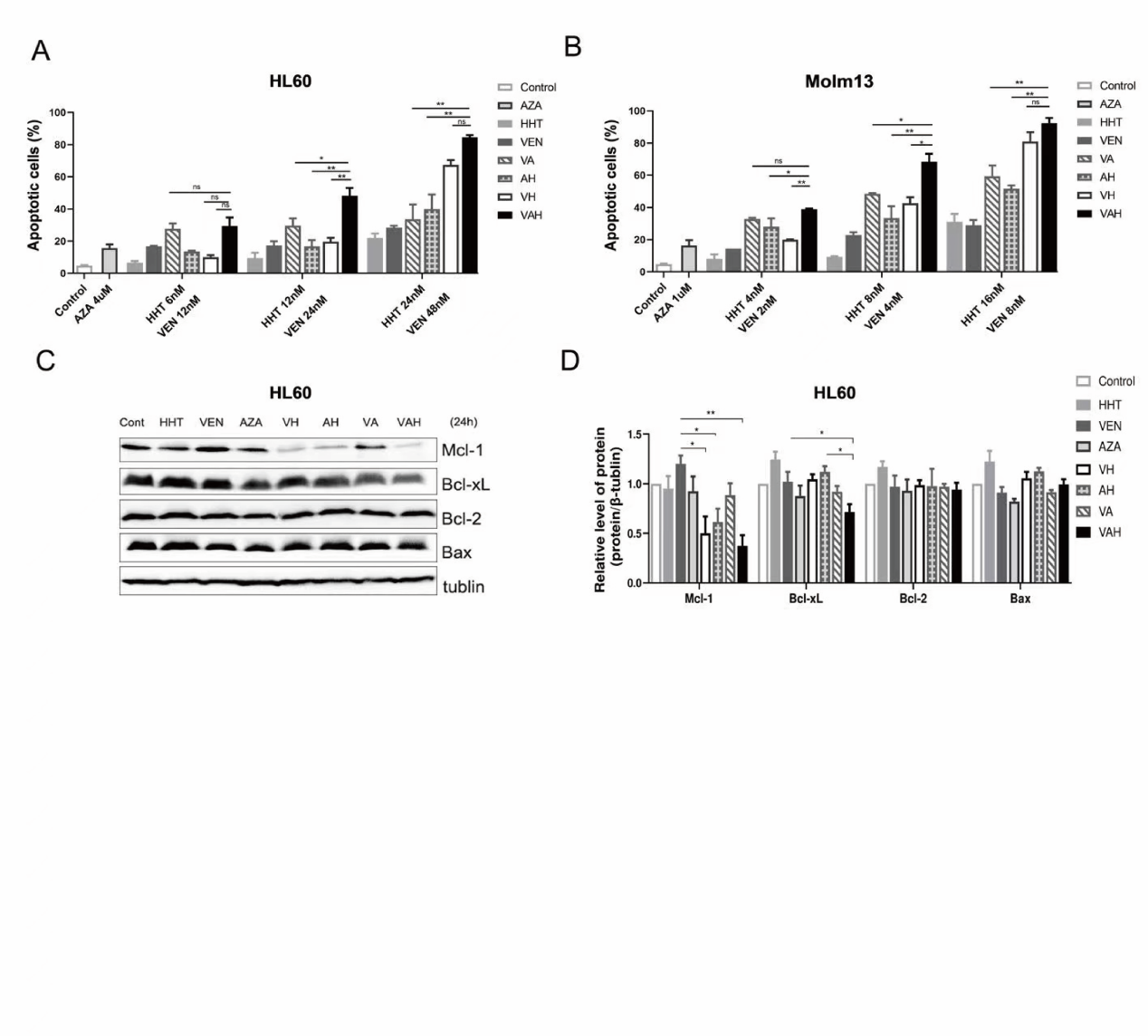


**Supplemental Figure 1. Homoharringtonine enhanced the pro-apoptotic effect of** **venetoclax with or without** **azacitidine in AML cell lines.** Homoharringtonine strengthened the anti-leukemia effect of venetoclax. Combination of homoharringtonine with venetoclax presented a synergistic pro-apoptotic effect in HL60 and Molm13 cells (A,B). Combination of homoharringtonine with venetoclax with or without azacytidine significantly decreased MCL1 expression in HL60 cells treated. Western blot analysis of the expression of MCL1, BCL-2, BCL-XL, and BAX in HL60 cells treated with homoharringtonine, venetoclax, and azacitidine as indicated. Quantification of the gray level of different western bands,showing as a ratio of testing band versus control band (C-D). VH, venetoclax plus homoharringtonine; AH, azacitidine plus homoharringtonine; VA, venetoclax plus azactidine; VAH, homoharringtonine combined with venetoclax plus azacitidine (*p<0·05; **p<0·01; ***p<0·001).

S2


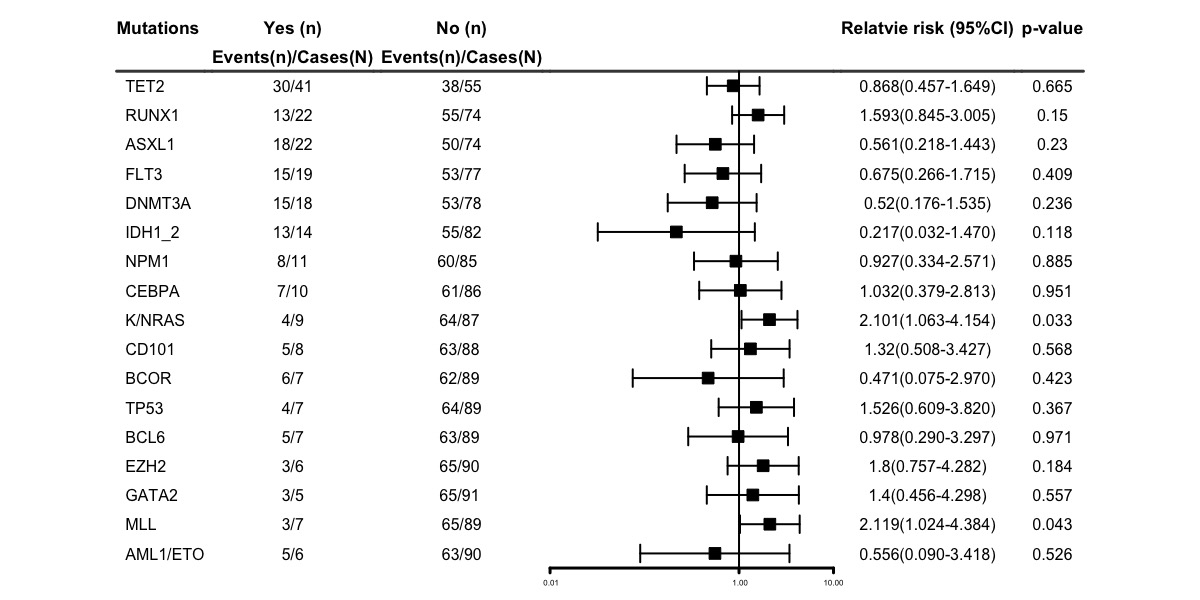


**Supplemental Figure 2**. **Molecular mutation subgroup analysis of CRc.** The relative risk of CRc between subgroups and corresponding 95% confidence Interval (CI) were calculated using binomial regression.

S3


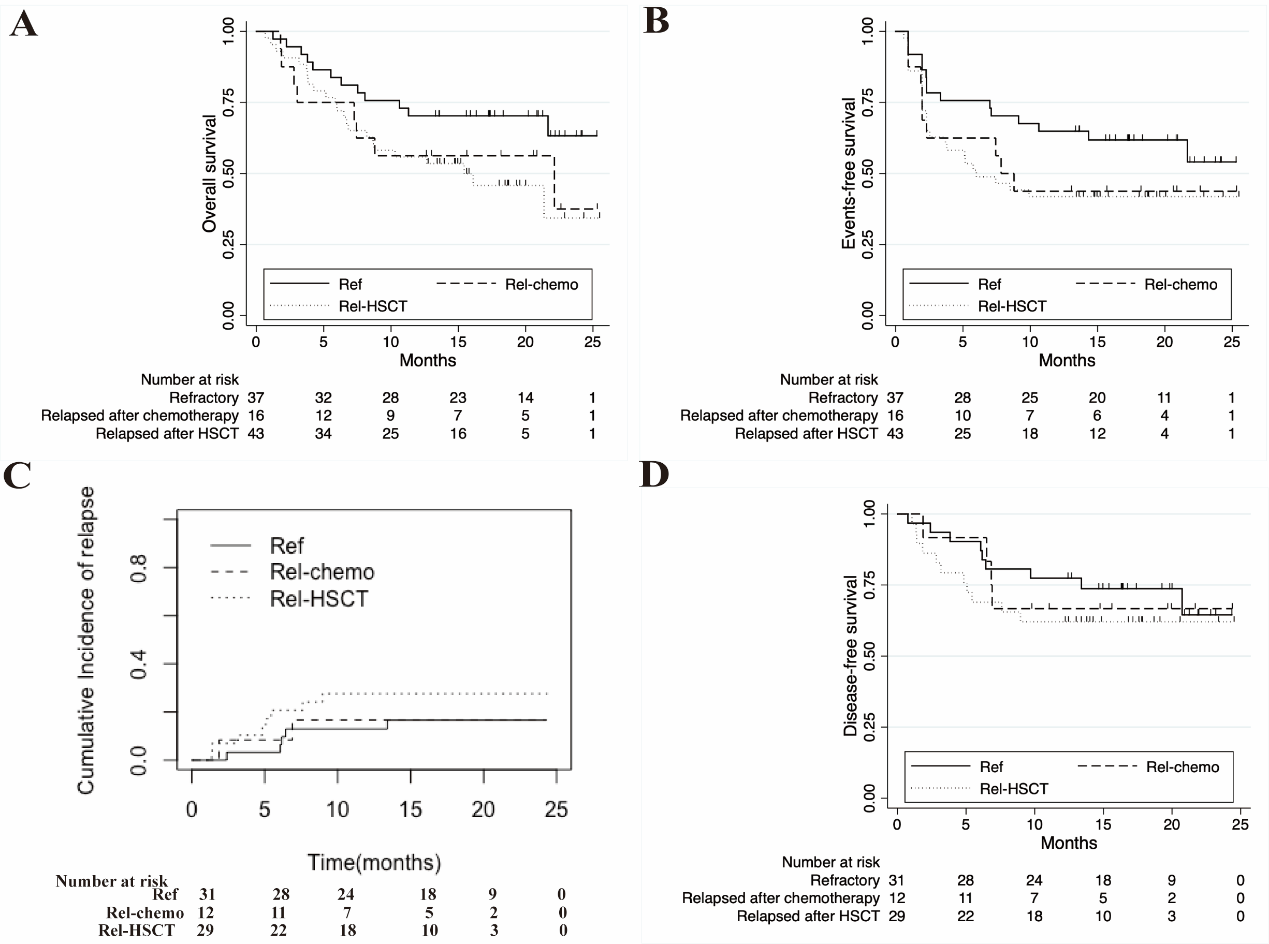


**Supplemental Figure 3. Cumulative incidence of overall survival (A), event-free survival (B), relapse(C) and disease-free survival (D) in disease subgroup.**

Ref, refractory. Rel-chemo, relapsed after chemotherapy. Rel-HSCT, relapsed after HSCT. HSCT, hematopoietic stem cell transplantation.

**S4**


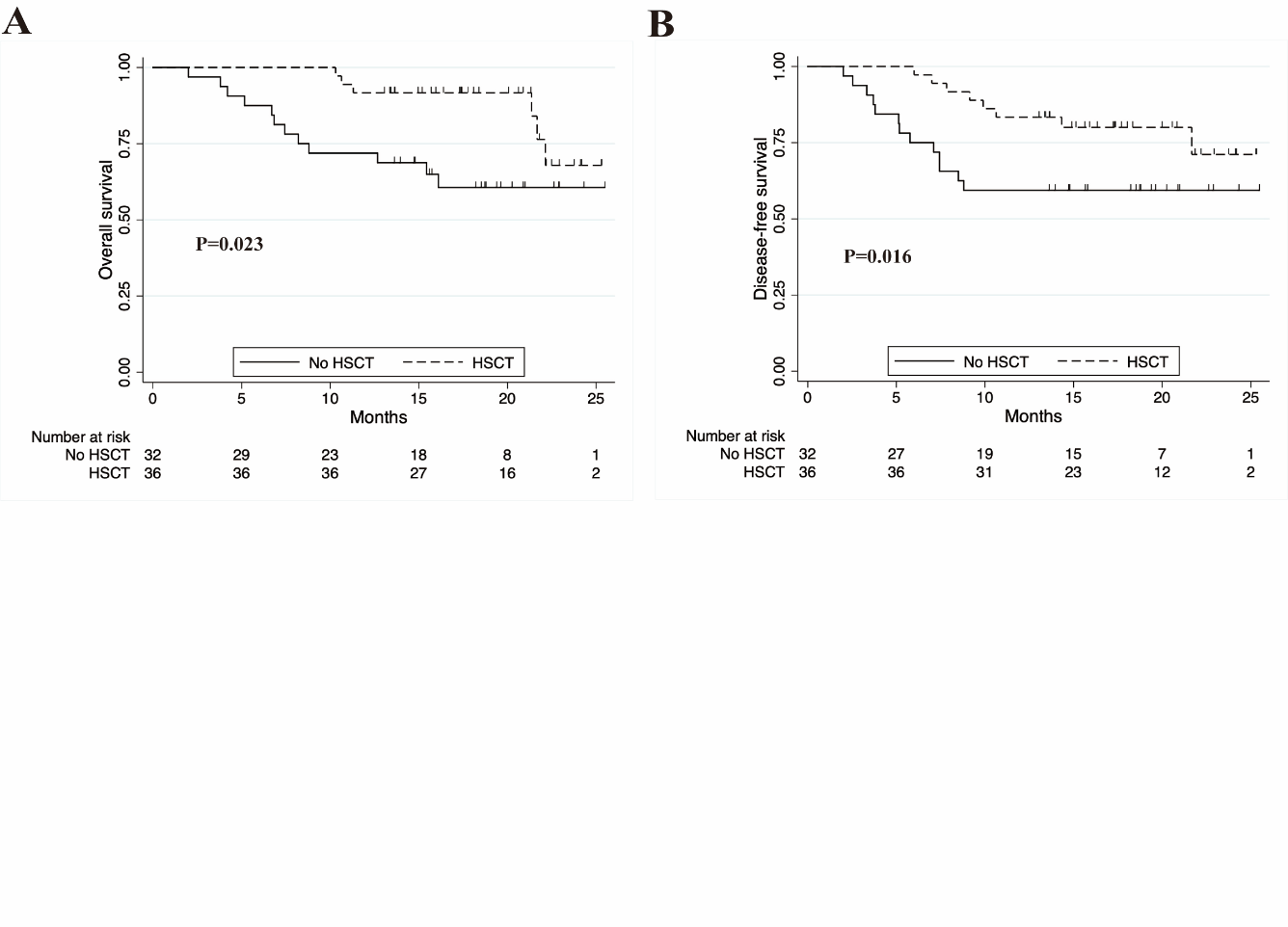


**Supplemental Figure 4. Among patients reached CRc, cumulative incidence of overall survival (A) and disease-free survival (B) in patients who received or did not receive allo-HSCT.**

**S5**


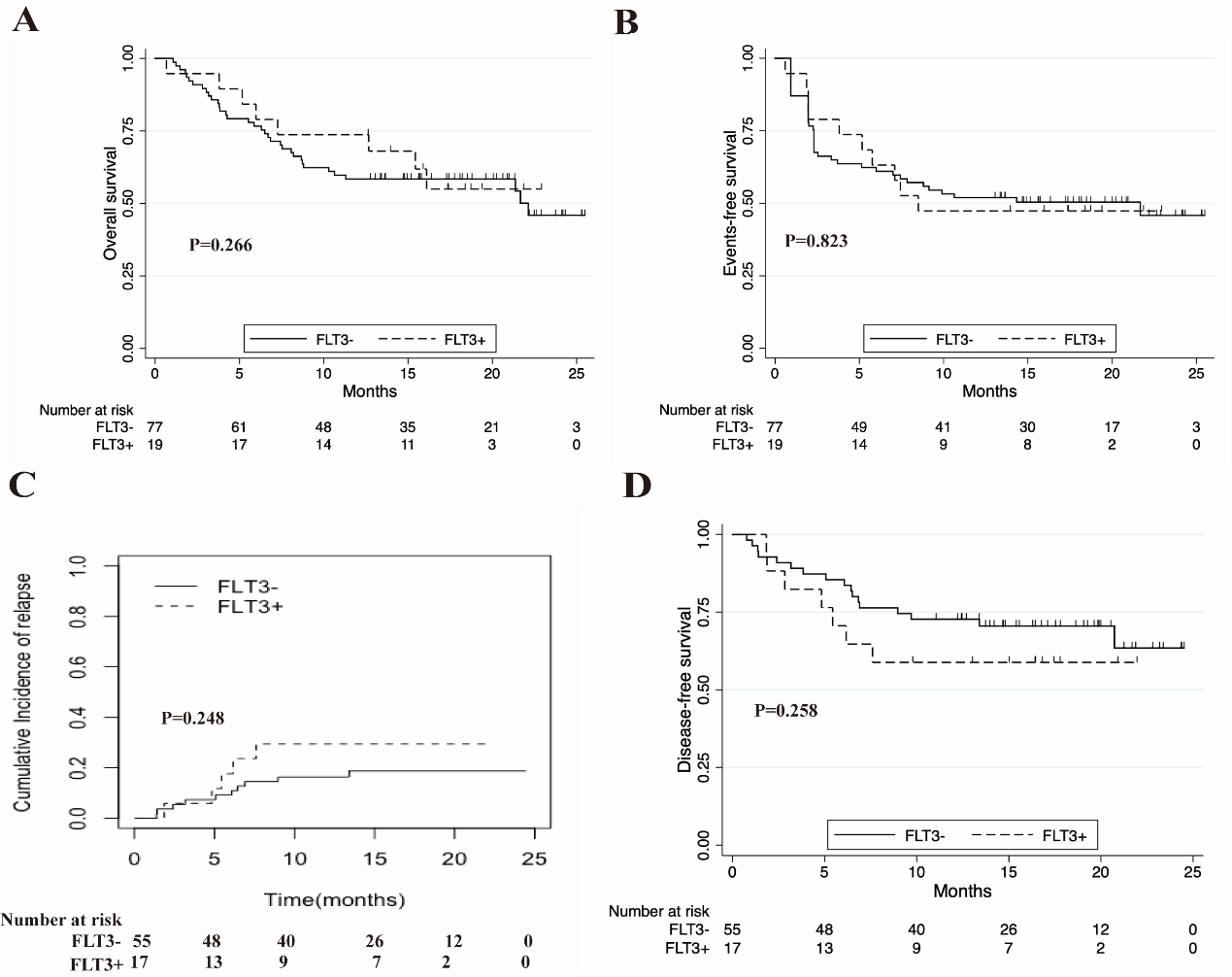


**Supplemental Figure 5. Cumulative incidence of overall survival (A), event-free survival (B), relapse(C) and disease-free survival (D) among** **patients without FLT3^mut^ and patients with FLT3^mut^**

**Supplemental Table 1. CRc rates of cytogenetic and molecular subgroups**

| **Subgroups** | **CRc (CR+CRi), No. (% [95% CI])** | | | |
| --- | --- | --- | --- | --- |
|  | **All（n=96）** | **Refractory AML**  **(n=37)** | **Relapsed after chemotherapy AML**  **(n = 16)** | **Relapsed after allo-HSCT AML**  **(n =43 )** |
| **ELN classification** |  |  |  |  |
| **Favorable** | **16(94.1 [66.0-99.2])** | **7(100)** | **2(100)** | **7(87.5 [41.1-98.6])** |
| **Intermediate** | **14(63.6 [41.6-81.2])** | **7(70.0 [34.8-90.1])** | **3(60.0 [14.4-93.1])** | **4(57.1 [20.1-87.6])** |
| **Adverse** | **38(66.7 [53.3-77.8])** | **15(75.0 [50.6-89.8])** | **5(55.6 [21.5-85.1])** | **18(64.3 [44.4-80.2])** |
| **Cytogenetics‡** |  |  |  |  |
| **Favorable** | **6 (100 )** | **4 (100 )** | **0(0)** | **2 (100 )** |
| **Intermediate** | **41(77.4 [63.9-86.8])** | **16(88.9 [62.6-97.5])** | **7(70.0 [33.1-91.7])** | **18(72.0 [50.7-86.6])** |
| **Poor** | **15(55.6 [36.3-73.2])** | **8(66.7 [35.4-88.0])** | **1(33.3 [2.0-92.6])** | **6(50.0 [22.8-77.2])** |
| **Unknown** | **6(60.0 [28.0-85.3])** | **1(33.3 [2.3-91.3])** | **2(66.7 [7.6-98.0])** | **3(75.0 [16.9-97.8])** |
| **Molecular abnormalities** |  |  |  |  |
| **NPM1** | **8(72.7 [39.4-91.6])** | **3(100)** | **1(50.0 [1.4-98.6])** | **4(66.7 [22.8-93.1])** |
| **AML1-ETO** | **5(83.3 [31.6-98.2])** | **3(75.0 [16.8-97.8])** | **0(0)** | **2(100)** |
| **CEBPA** | **7(70.0 [35.5-90.8])** | **2(66.7 [8.7-97.7])** | **1(50.0 [1.4-98.6])** | **4(80.0 [24.3-98.0])** |
| **TET2** | **30(73.2 [57.3-84.7])** | **12(92.3 [57.1-99.1])** | **4(57.1 [18.7-88.6])** | **14(66.7 [43.4-83.9])** |
| **DNMT3A** | **15(83.3 [57.9-94.8])** | **8(88.9 [45.0-98.7])** | **2(100)** | **5(71.4 [28.8-93.9])** |
| **IDH1/2**  **FLT3**  **ASXL1**  **RUNX1**  **TP53**  **MLL** | **13(92.9 [60.5-99.1])** | **8(100)** | **2(66.7 [7.6-98.0])** | **3(100)** |
| **FLT3** | **15(78.9 [54.3-92.2])** | **7(100)** | **0(0)** | **8(80.0 [42.7-95.6])** |
| **ASXL1** | **18(81.8 [59.4-93.3])** | **7(100)** | **2(50.0 [7.9-92.1])** | **9(81.8 [46.2-95.9])** |
| **RUNX1** | **13(59.1 [37.4-77.7])** | **5(83.3 [30.5-98.3])** | **2(66.7 [7.6-98.0])** | **6(46.2 [21.0-88.9])** |
| **TP53** | **4(57.1 [20.6-87.3])** | **2(50.0 [8.8-91.2])** | **0(0)** | **2(66.7 [8.8-97.6])** |
| **MLL** | **3(42.9 [12.7-79.4])** | **2(100)** | **1(33.3 [2.0-92.6])** | **0(0)** |
| **EZH2** | **3(50.0 [14.5-85.5])** | **0(0)** | **1(100)** | **2(50.0 [8.9-91.1])** |
| **BCL6** | **5(71.4 [29.4-93.8])** | **1(100)** | **0(0)** | **4(80.0 [24.3-98.0])** |
| **BCOR** | **6(85.7 [37.2-98.4])** | **5(83.3 [30.5-98.3])** | **0(0)** | **1(100)** |
| **GATA2** | **3(60.0 [16.5-91.9])** | **1(100)** | **0(0)** | **2(50.0 [8.9-91.1])** |
| **RAS** | **4(44.4 [16.3-76.7])** | **3(50.0 [14.0-86.0])** | **0(0)** | **1(50.0 [1.7-98.3])** |
| **CD101** | **5(62.5 [26.1-88.7])** | **2(100)** | **0(0)** | **3(60.0 [16.1-92.2])** |

Abbreviations: CRc, composite complete remission; CR, complete remission; CRi, CR with incomplete hematological recovery; ELN, European Leukemia Net; AML, acute myeloid leukemia; Allo-HSCT, allogeneic hematopoietic stem cell transplantation.

**Supplemental Table 2. Univariate and multivariate analyses for the risk factors of overall survival**

|  | **Overall survival** | | | |
| --- | --- | --- | --- | --- |
| **Characteristics** | **Univariable** | | **Multivariable** | |
|  | **HR (95%CI)** | ***P*** | **HR (95%CI)** | ***P*** |
| **Age***  **>45 vs** ≤**45** | **0.76(0.41-1.40)** | **0.378** |  |  |
| **Gender**  **Female vs Male** | **1.11(0.61-2.03)** | **0.726** |  |  |
| **AML status**  **Refractory vs Relapsed after chemotherapy**  **Refractory vs Relapsed after Allo-HSCT**  **Relapsed after chemotherapy vs Relapsed after Allo-HSCT** | **1.42(1.01-1.99)**  **0.55(0.22-1.34)**  **0.49(0.24-0.98)**  **0.89(0.40-1.99)** | **0.046**  **0.185**  **0.044**  **0.777** |  |  |
| **ELN classification**  **Intermediate vs Favorable**  **Adverse vs Favorable**  **Adverse vs Intermediate** | **1.60(1.03-2.49)**  **2.26(0.69-7.37)**  **2.95(1.04-8.39)**  **1.31(0.62-2.76)** | **0.037**  **0.177**  **0.042**  **0.483** | **1.53(0.82-2.88)** | **0.184** |
| **MRD**  **Negative vs positive** | **0.31(0.11-0.82)** | **0.018** | **0.35(0.13-0.93)** | **0.035** |
| **Bridge to allo-HSCT**  **Yes vs No** | **0.24(0.11-0.49)** | **<0·001** | **0·36(0·13–0·98)** | **0·046** |

Abbreviations: Allo-HSCT, allogeneic hematopoietic stem cell transplantation; ELN, European Leukemia Net; MRD, minimal residual disease. *Cutoffs were the median value.

|  |
| --- |
