# Supplementary material for: Venetoclax Combined with Azacitidine and Homoharringtonine in Relapsed/Refractory AML: A Multicenter, Phase 2 Trial
Source: J Hematol Oncol. 2023 Apr 29;16:42. doi: 10.1186/s13045-023-01437-1 (PMC10149010; doi:10.1186/s13045-023-01437-1)
Supplement: Supplementary file 2 — Additional file 2. Clinical study protocol. [file 13045_2023_1437_MOESM2_ESM.pdf]

CLINICAL STUDY PROTOCOL

Protocol Title: Venetoclax combined with azacitidine plus homoharringtonine in relapsed/refractory acute myeloid leukemia： A multicenter, phase 2 trial

Protocol number: NCT04424147

Study regimens: VAH

Study Phase: 2

Indication: Salvage therapy of relapsed/refractory acute myeloid leukemia

Applicant Institution: Nanfang Hospital, Southern Medical University

Principal institution: Nanfang Hospital, Southern Medical University

INVESTIGATOR'S STATEMENT

I have received and completely reviewed the following protocol (NCT04424147), including all appendices:

As Principal Investigator, I understand and agree to conduct this clinical study as described and will comply with the ethical and regulatory considerations delineated herein.

Study Title

Venetoclax combined with azacitidine plus homoharringtonine in relapsed/refractory acute myeloid leukemia： A multicentre, phase 2 trial

Principal Investigator Signature and Contact Information

Principal Investigator (print) ______________________________

Principal Investigator (signature) ______________________________

Date of Signature ______________________________

Institution/Affiliation ______________________________

City, Province, Country ______________________________

Study Synopsis

**Study title**	Venetoclax combined with azacitidine plus homoharringtonine in relapsed/refractory acute myeloid leukemia：A multicentre, phase 2 trial
**Protocol number**	NCT04424147
**Indication**	Salvage therapy of relapsed (R) /refractory (R) acute myeloid leukemia (AML)
**Study phase**	2
**Study applicant**	Nanfang Hospital, Southern Medical University
**Study centers**	Ten study centers consist of :<br>1. Nanfang Hospital, Southern Medical University;<br>2. Shenzhen Second People's Hospital;<br>3. Sun Yat-Sen Memorial Hospital, Sun Yat-Sen University;<br>4. Shenzhen Hospital of Southern Medical University;<br>5. Seventh Affiliated Hospital of Sun Yat-Sen University;<br>6. Maoming People's Hospital;<br>7. The First People's Hospital of Chenzhou;<br>8. Peking University Shenzhen Hospital;<br>9. Zhongshan City People's Hospital;<br>10. Shanghai Ninth People’s Hospital.
**Number of subjects planned**	Approximately 96 subjects will be enrolled.
**Study duration**	Estimated to be 2 years
Objectives	
**Primary**	The primary endpoint is composite complete remission (CRc) rate

**objective:**	after 2 cycles of trial therapy.
**Secondary objectives:**	The secondary endpoints are safety, overall survival (OS), event-free survival (EFS), disease-free survival (DFS), and relapse. OS was defined as the time from treatment until death or censored at the last follow-up. EFS was defined as the time from treatment until documented failure to achieve CRc, relapse after CRc, or death from any cause, whichever occurred first. DFS was defined as the time from the date of CRc until relapse, death from any cause, or censored at the last follow-up. Data for each patient are censored at the date of the last visit or the date on which the patient is last known to be alive.
**Study design**	This is a prospective, multi-center, phase 2 clinical study that investigate the efficacy and tolerability of venetoclax (VEN) combined with azacitidine (AZA) plus homoharringtonine (HHT) (VAH) regimen in patients with R/R AML. Approximately 96 subjects will be enrolled.<br>VAH regimen: VEN begins at 100 mg on day 1 and increases stepwise over 3 days to reach the target dose of 400 mg (100 mg, 200 mg, 400 mg); dosing is continued at 400 mg per day from day 4 through day 14; AZA (75 mg/m^2) is administered subcutaneously on days 1-7, and HHT (1 mg/m^2) on days 1-7.<br>VEN dose will be reduced by at least 50% in the patients receiving concomitant moderate or strong CYP3A4 inhibitors (eg, azole antifungals). FLT3 inhibitors are allowed in the patients with FLT3 mutations. Donor lymphocyte infusion (DLI) is administered at day 15 after the initiation of the trial therapy for the relapse patients after allogeneic haematopoietic stem-cell transplantation (allo-HSCT). Once acquiring CRc, the patients are recommended to

	receive allo-HSCT if donors are available. If donors are unavailable, patients will receive one course of original therapy again and sequential cytarabine-based consolidation therapy. If patients do not obtain CRc after two courses of the trial therapy, they will proceed to allo-HSCT if donors are available, and patients receive other salvage therapy based on patients' personal preferences after full discussion with physicians if donors are unavailable. In addition, for the patients undergoing allo-HSCT, sorafenib maintenance post-transplantation was recommended regardless of FLT3 being mutated or not.<br>Response assessments: Bone marrow is evaluated on cycle 1 day 28 and again 1-2 weeks after hematological recovery if the day 28 bone marrow is aplastic. Subsequent bone marrow evaluations are done before and after cycles 2, and then as clinically needed. Morphological, cytogenetic and minimal residual disease (MRD) assessments are done during each bone marrow assessment. Complete remission (CR) is defined as an absolute neutrophil count of more than 1000 cells per cubic millimeter, a platelet count of more than 100,000 per cubic millimeter, red-cell transfusion independence, and bone marrow with less than 5% blasts. Complete remission with incomplete hematologic recovery (CRi) is defined as all the criteria for CR, except for neutropenia (absolute neutrophil count, ≤1000 per cubic millimeter) or thrombocytopenia (platelet count, ≤100,000 per cubic millimeter). Partial remission (PR) is a minimal residual disease of 5-25% with a greater than 50% decrease in leukemic blast percentage. Non-remission (NR) is defined as a failure to obtain CRc or PR. CRc comprised CR and CRi, and overall response rate (ORR) comprised CRc and PR. The MRD level of 0.01% is used as a threshold to distinguish MRD positive (MRD^+)

	from MRD negative (MRD$^-$).
**Inclusion criteria**	Subjects eligible for enrolment in this study must meet all of the following criteria:<br>1. Patients with relapsed/refractory AML<br>Refractory AML is defined as no CRc and a reduction in bone marrow blasts of less than 50% after one cycle or no CRc after two cycles. Relapsed AML is defined as recurrence of blasts in the peripheral blood or bone marrow blasts≥5% or development of extramedullary disease after achieving CRc.<br>2. Age 18 to 65 years old with Eastern Cooperative Oncology Group (ECOG) performance status of 0-2<br>3.Sign informed consent form, have the ability to comply with study and follow-up procedures
**Exclusion criteria**	Subjects meeting any of the following criteria are ineligible for this study:<br>1. Acute promyelocytic leukemia (AML subtype M3)<br>2. Previous exposure to the treatment of VEN-based regimen<br>3.Cardiac dysfunction (particularly congestive heart failure, unstable coronary artery disease and serious cardiac ventricular arrhythmias requiring antiarrhythmic therapy)<br>4. Respiratory failure (PaO2 ≤60mmHg)<br>5. Hepatic abnormalities (total bilirubin ≥2 times the upper limit of normal [ULN], alanine aminotransferase (ALT) or aspartate aminotransferase (AST) ≥2 times the ULN)<br>6. Renal dysfunction (creatinine ≥2 times the ULN or creatinine clearance rate < 30 mL/min)<br>7. ECOG performance status 3, 4 or 5<br>8. Substantial history of neurological, psychiatric, endocrine, metabolic, immunological, or any other medical condition not

	suitable for the trial (investigators' decision)<br>9. Active acute or chronic graft-versus-host disease (GVHD). Active acute GVHD or chronic GVHD is defined as GVHD requiring either at least 1 mg/kg per day of prednisone (or equivalent) or treatment beyond systemic corticosteroids.<br>10. Patients with pregnancy<br>11. Uncontrolled active infection<br>12. Clinically significant coagulation abnormalities
**Study treatment**	VAH regimen: VEN begins at 100 mg on day 1 and increases stepwise over 3 days to reach the target dose of 400 mg (100 mg, 200 mg, 400 mg); dosing is continued at 400 mg per day from day 4 through day 14, AZA (75 mg/m²) is administered subcutaneously on days 1-7, and HHT (1 mg/m²) on days 1-7.<br>VEN dose will be reduced by at least 50% in the patients receiving concomitant moderate or strong CYP3A4 inhibitors (eg, azole antifungals). FLT3 inhibitors are allowed in the patients with FLT3 mutations. DLI is administered at day 15 after the initiation of the trial therapy for the relapse patients after allo-HSCT. Once acquiring CRc, the patients are recommended to receive allo-HSCT if donors are available. If donors are unavailable, patients will receive one course of original therapy again and sequential cytarabine-based consolidation therapy. If patients do not obtain CRc after two courses of the trial therapy, they will proceed to allo-HSCT if donors are available, and patients receive other salvage therapy based on patients' personal preferences after full discussion with physicians if donors are unavailable. In addition, for the patients undergoing allo-HSCT, sorafenib maintenance post-transplantation was recommended regardless of FLT3 being mutated or not.
**Sample size**	The sample size calculation for the trial was based on the

**determination**	assumption that the VAH regimen would achieve a higher CRc rate compared with historical CRc rate of 45% (on the basis of the venetoclax in combination with HMAs study in R/R AML). To identify a 15% absolute improvement in CRc with VAH regimen, a total of 87 patients was required to provide the study with a significance level of 5% and a power of 80%. After adjusting for a 10% dropout, the total planned sample size was 96 patients. The sample size calculation was done using PASS software, version 11·0.
**Statistical analysis**	Statistical analysis is performed based on the intent-to-treat (ITT) population, which includes all subjects<br>● The primary endpoint is CRc rate after 2 cycles of trial treatment.<br>● The secondary endpoints are safety, overall survival (OS), event-free survival (EFS), disease-free survival (DFS) and relapse.<br>OS is defined as the time from treatment until death or censored at the last follow-up.<br>EFS is defined as the time from treatment until documented failure to achieve CRc, relapse after CRc, or death from any cause, whichever occurred first.<br>DFS is defined as the time from the date of CRc until relapse, death from any cause, or censored at the last follow-up.<br>Data for each patient are censored at the date of the last visit or the date on which the patient was last known to be alive.<br>● Adverse events are recorded up to 4 weeks after the discontinuation of the trial therapy, and are graded according to the National Cancer Institute Common Terminology Criteria for Adverse Events (CTCAE) version 4.0. The study adjudication

	committee（consisting of experts in hematology, infection, pathology, pharmacy, and statistics） judge whether adverse events are treatment-related or non-treatment-related. Serious adverse events are those that resulted in death, disability, or incapacity, are life-threatening or judged an important medical event, or required hospitalization or prolongation of existing hospitalization.<br>● The descriptive analysis of patient characteristics included median and interquartile range (IQR) for continuous variables, and absolute and relative frequencies for categorical variables. The time-to-event endpoints including OS, EFS and DFS were analyzed by the Kaplan-Meier method and compared using the log-rank tests. The corresponding hazard ratio (HR) and 95% CI were estimated using the Cox proportional hazards model. The cumulative incidences of relapse were calculated by accounting for competing risks, and non-relapse mortality was a competing risk for relapse. The comparison of the cumulative incidence in the presence of a competing risk was done using the Fine and Gray model.[23] All variables in the Cox models were tested for proportional hazards assumption. Factors that were significant at the 0.1 level from the univariable model were included in the multivariable model. All statistics were analyzed in software R version 4.1.0 (R Development Core Team, Vienna, Austria) or Stata version 15.1 (StataCorp 4905 Lakeway Dr College Station, TX77845, USA) or SPSS version 22.0 (SPSS, Chicago, IL). All statistical tests were two-tailed with a significance level of 0.05.

TABLE OF CONTENTS

PAGE

Clinical Study Protocol..1
Investigator's Statement...2
Study Synopsis..3
Table of Contents...11
Abbreviations...13
1. Introduction...15
2. Study objectives...16
2.1 Primary Objective.. 16
2.2 Secondary Objectives..16
3. Study Design...16
4. Subject Selection Criteria.. 19
4.1 Subject Selection Criteria...19
4.1.1 Number of Subjects..19
4.1.2 Inclusion Criteria..19
4.1.3 Exclusion Criteria...20
4.2. Withdrawal Criteria...21
5. Study Procedures...21
5.1 Screening...21
5.2 Study Treatment..22
5.3 Follow-up..22
6. Efficacy Assessments...23
6.1 Definitions...23
6.2 Primary Efficacy Endpoint...23
6.3 Secondary Efficacy Endpoints..23
6.4 Schedule and methods of Efficacy Assessments...24
7. Safety Evaluation...24
7.1 Medical History...24
7.2 Vital Signs and Physical Examination..24
7.3 Clinical Symptoms...25
7.4 Clinical Laboratory Evaluations..25
8. Adverse Events and Serious Adverse Events (SAEs)...26
8.1 Definitions...26
8.1.1 Adverse Events..26
8.1.2 Serious Adverse Events..26
8.2 Assessment of Severity...27
8.3 Assessment of Causality...27
8.4 Recording and Reporting AEs and SAEs...27
9. Rules of Withdrawal...28
9.1 Subjects Withdraw from the Study...28
9.2 Premature Termination of the Study...28
10. Rules of Follow-Up..29
10.1 Follow-up Period..29

10.2 Visit Scheduling....29
10.3 Contents....29
11. Data Analysis and Statistical Considerations....29
11.1 Hypotheses....29
11.2 Study Design Considerations....29
11.2.1 Sample Size Assumptions....30
11.2.2 Primary Efficacy Endpoint....30
11.2.3 Secondary Efficacy Endpoints....30
11.3 Data Analysis Considerations....30
11.3.1 Analysis Population....30
11.3.2 Analysis Plan....31
11.3.2.1 Baseline Data....31
11.3.2.2 Analysis of Efficacy....31
11.3.2.3 Analysis of Safety....31
12. Materials for the Study....32
13. Ethical Considerations....32
13.1 Responsibility of Investigators....32
13.2 Informed Consent Process....32
13.3 Good Clinical Practice....32
13.4 Protection of Subjects' Personal Data....33
14. Administrative Requirements....33
15. References....33

Abbreviations

R/R	Relapsed/refractory
AML	Acute myeloid leukemia
CR	Complete remission
CRi	Complete remission with incomplete hematologic recovery
PR	Partial remission
NR	Non-remission
ORR	Overall response rate
CRc	Composite complete remission
OS	Overall survival
DFS	Disease-free survival
VEN	Venetoclax
AZA	Azacitidine
HHT	Homoharringtonine
VAH	VEN+AZA+HHT
DLI	Donor lymphocyte infusion
Allo-HSCT	Allogeneic haematopoietic stem-cell transplantation
MRD	Minimal residual disease
ECOG	Eastern Cooperative Oncology Group
ULN	Upper limit of normal
ALT	Alanine aminotransferase
AST	Aspartate aminotransferase
GVHD	Graft-versus-host disease
HMAs	Hypomethylating agents
ITT	Intent-to-treat
IQR	Interquartile range
HR	Hazard ratio
CI	Confidence interval

BCL-2	Anti-apoptotic B-cell lymphoma 2
MCL1	Myeloid-cell leukemia 1
BM	Bone marrow
PB	Peripheral blood
CRF	Case report form
SAEs	Serious adverse events
AEs	Adverse events
CTCAE	Common Terminology Criteria for Adverse Events
GCP	Good clinical practice

1. Introduction

Acute myeloid leukemia (AML) is one of the major diseases threatening human health. Refractory and relapse (R/R) are the most important reason for the failure of AML treatment, which eventually lead to the progression of more than one half of AML patients. At present, the low remission rate, short time of survival and lack of well-accepted and effective treatment are the main problem of R/R AML. The exact mechanism of drug resistance in R/R AML is not yet up until now completely clear. Clinical trials are the first choice for the treatment of R/R AML which is recommended in the National Comprehensive Cancer Network (NCCN) guideline to improve the efficacy of this group of patients. Therefore, the current difficulty and focus of the research in R/R AML is to further clarify the mechanism of drug resistance and explore a safe and effective therapy.

The high expression of anti-apoptotic B-cell lymphoma 2 (BCL-2) is an important reason for drug resistance in AML [1-4]. Combination of BCL-2 inhibitor venetoclax (VEN) with hypomethylating agents (HMAs) or low dose cytarabine have achieved good results in the elderly or unfit-AML patients, and approved by FDA and recommended by NCCN guidelines for the recommended treatment of elderly and unfit-AML [5-7]. Also, R/R AML patients have achieved encouraging results with the salvage treatment of VEN-based therapy, but the CR rate is not as high as expected, and the duration of complete remission is short [8-9]. Furthermore, the curative effect in the patients relapsed after allo-HSCT is very poor. Therefore, how to improve the efficacy of VEN-based regimen and overcome VEN resistance are an urgent problem to be solved.

High expression of myeloid-cell leukemia 1 (MCL1) and BCL-XL is recognized as an important factor for VEN resistance [10-11]. Studies [12-14] have revealed that inhibition of MCL-1 expression can improve the anti-leukemia effect of VEN. Homoharringtonine (HHT), widely used in the treatment of hematological malignant tumors in China since the 1970s. HHT has also received more widespread attention and approved by the FDA in 2010 for the treatment of R/R CML [15] and AML [16], is

reported to inhibit the expression of MCL-1 and BCL-XL in leukemic cells. The previous study [17] had reported that HHT combined with VEN could further inhibit the expression of MCL-1 and BCL-2 in lymphoma cells and increase the anti-lymphoma effect, suggesting that HHT might enhance the anti-tumor effect of VEN. Our preliminary study showed that HHT enhanced the anti-leukemia effect of VEN with or without AZA through deeper inhibition of MCL1, BCL-XL in AML cell lines. To further optimise salvage therapy for R/R AML patients, we set up a multicenter, phase 2 trial to investigate the efficacy and tolerability of VAH regimen in patients with R/R AML.

2. Study objectives

2.1 Primary Objective

The primary endpoint is CRc rate after 2 cycles of trial treatment.

2.2 Secondary Objectives

The secondary endpoints are safety, overall survival (OS), event-free survival (EFS), disease-free survival (DFS), and relapse. OS is defined as the time from treatment until death or censored at the last follow-up. EFS is defined as the time from treatment until documented failure to achieve CRc, relapse after CRc, or death from any cause, whichever occurred first. DFS is defined as the time from the date of CRc until relapse, death from any cause, or censored at the last follow-up.. Data for each patient are censored at the date of the last visit or the date on which the patient is last known to be alive.

3. Study Design

This is a prospective, multi-center, phase 2 clinical study to investigate the efficacy and tolerability of VAH regimen in patients with R/R AML. Approximately 96 subjects will be enrolled to receive VAH regimen for salvage therapy. The study design is illustrated in Figure 1

Subjects with R/R AML will be screened for eligibility. Medical history evaluation, vital sign, physical examination, ECOG performance status, blood and urine sampling

for laboratory tests, electrocardiogram, chest imaging examination as well as bone marrow (BM) assessment will be performed to determine study eligibility.

After enrolment, patients will be assigned to the trial therapy. For VAH treatment, VEN begins at 100 mg on day 1 and increases stepwise over 3 days to reach the target dose of 400 mg (100 mg, 200 mg, 400 mg); dosing is continued at 400 mg per day from day 4 through day 14； AZA (75 mg/m²) is administered subcutaneously on days 1-7, and HHT (1 mg/m²) on days 1-7. VEN dose will be reduced by at least 50% in the patients receiving concomitant moderate or strong CYP3A4 inhibitors (eg, azole antifungals). FLT3 inhibitors are allowed in the patients with FLT3 mutations. Donor lymphocyte infusion (DLI) is administered at day 15 after the initiation of the trial therapy for the relapse patients after allo-HSCT. Once acquiring CRc, the patients are recommended to receive allo-HSCT if donors are available. If donors are unavailable, patients will receive one course of original therapy again and sequential cytarabine-based consolidation therapy. In addition, for the patients undergoing allo-HSCT, sorafenib maintenance post-transplantation was recommended regardless of FLT3 being mutated or not. If patients do not obtain CRc after two courses of the trial therapy, they will proceed to allo-HSCT if donors are available, and patients receive other salvage therapy based on patients' personal preferences after full discussion with physicians if donors are unavailable.

For response assessments, bone marrow is evaluated on cycle 1 day 28 and again 1-2 weeks after hematological recovery if the day 28 bone marrow is aplastic. Subsequent bone marrow evaluations are done before and after cycles 2, and then as clinically needed. Morphological and MRD assessments are done during each bone marrow assessment. CR is defined as an absolute neutrophil count of more than 1000 cells per cubic millimeter, a platelet count of more than 100,000 per cubic millimeter, red-cell transfusion independence, and bone marrow with less than 5% blasts. CRi is defined as all the criteria for CR, except for neutropenia (absolute neutrophil count, ≤1000 per cubic millimeter) or thrombocytopenia (platelet count, ≤100,000 per cubic millimeter). Partial remission (PR) is a minimal residual disease of 5-25% with a greater than 50% decrease in leukemic blast percentage. Non-remission (NR) is defined as a failure to

obtain CRc or PR. CRc comprised CR and CRi, and overall response rate (ORR) includes CRc and PR. The MRD level of 0.01% is used as a threshold to distinguish MRD positive (MRD^{+}) from MRD negative (MRD^{-}).

The clinical data cutoff date is June 30, 2022. Criteria for removing patients from trial therapy includes the development of intolerable adverse events related to study treatment (determined by the treating physician), patient withdrew informed consent, and completion of the protocol therapy and evaluation period.

Figure 1 Study Schema

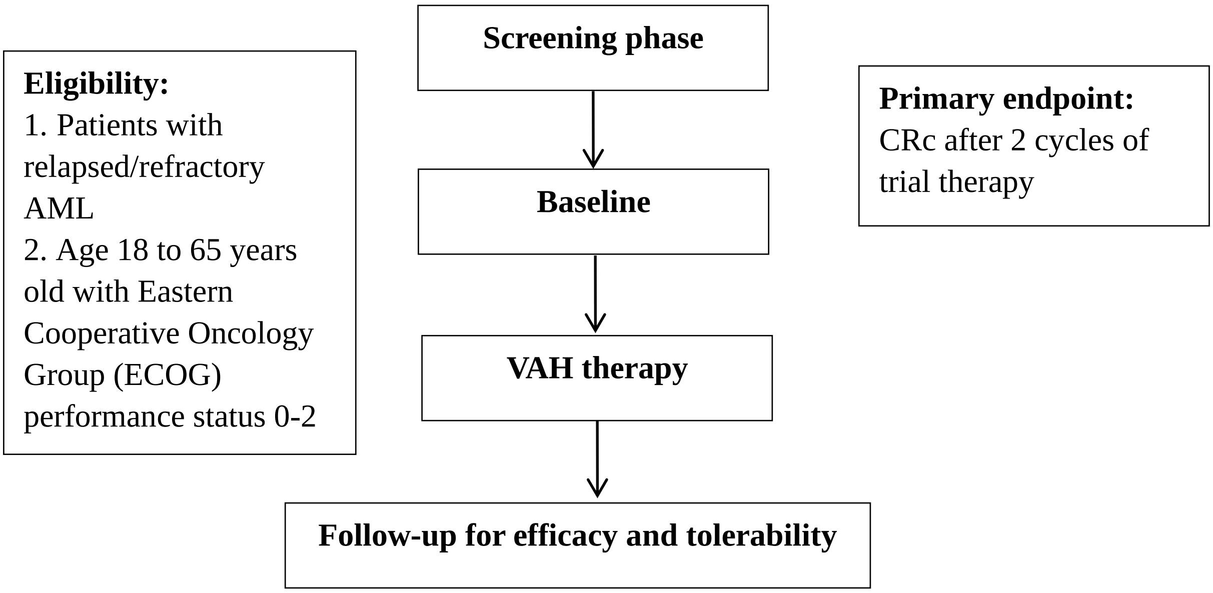

AML=acute myeloid leukemia; ECOG= Eastern Cooperative Oncology Group; CRc= composite complete remission; VAH=venetoclax+azacitidine+homoharringtonine.

4. Subject Selection Criteria

4.1 Subject Selection Criteria

4.1.1 Number of Subjects

Approximately 96 subjects will be enrolled to receive VAH regimen for salvage therapy.

4.1.2 Inclusion Criteria

Subjects eligible for enrolment in this study must meet all of the following criteria:

1. Patients with R/R AML

Refractory AML is defined as no CRc and a reduction in bone marrow blasts of less than 50% after one cycle or no CRc after two cycles. Relapsed AML is defined as recurrence of blasts in the peripheral blood or bone marrow blasts ≥5% or development of extramedullary disease after achieving CRc.

2. Age 18 to 65 years old with ECOG performance status of 0-2

3. Sign informed consent form, have the ability to comply with study and follow-up procedures.

4.1.3 Exclusion Criteria

Subjects meeting any of the following criteria are ineligible for this study:

1. Acute promyelocytic leukemia (AML subtype M3)

2. Previous exposure to the treatment of VEN-based regimen

3. Cardiac dysfunction (particularly congestive heart failure, unstable coronary artery disease and serious cardiac ventricular arrhythmias requiring antiarrhythmic therapy)

4. Respiratory failure (PaO2 ≤60mmHg)

5. Hepatic abnormalities (total bilirubin ≥2 times the upper limit of normal [ULN], ALT or AST ≥2 times the ULN)

6. Renal dysfunction (creatinine ≥2 times the ULN or creatinine clearance rate < 30 mL/min)

7. ECOG performance status 3, 4 or 5

8.Substantial history of neurological, psychiatric, endocrine, metabolic, immunological, or any other medical condition not suitable for the trial (investigators' decision)

9. Active acute or chronic graft-versus-host disease (GVHD). Active acute GVHD or chronic GVHD was defined as GVHD requiring either at least 1 mg/kg per day of prednisone (or equivalent) or treatment beyond systemic corticosteroids.

10. Patients with pregnancy

11. Uncontrolled active infection

12. Clinically significant coagulation abnormalities

4.2. Withdrawal Criteria

Subjects are free to withdraw consent and discontinue participation in the study at any time and without prejudice to future treatment. A subject's participation in the study may be discontinued at any time at the investigator's discretion. Justifiable reasons for a subject to be withdrawn from the study include:

1. Inability to fully comply with the study protocol
2. Unacceptable toxicity
3. Best interest of the subject based upon the investigator’s discretion
4. At the request of the study subject at any time and for any reason

Subjects will be followed up unless the informed consent is withdrawn. The reason for withdrawal from study participation and the date must be documented in the case report form (CRF). The investigator must complete the last visit, including vital signs, physical examination, laboratory tests, disease status and AE assessment, all of which must be documented in the CRF.

5. Study Procedures

5.1 Screening

Subjects with R/R AML will be screened for eligibility. Medical history evaluation, vital sign, physical examination, ECOG performance status, blood and urine sampling for laboratory tests, electrocardiogram, chest imaging examination as well as BM assessment will be performed to determine study eligibility, all of which must be performed ≤3 days prior to randomization.

5.2 Study Treatment

VAH salvage therapy

VEN begins at 100 mg on day 1 and increases stepwise over 3 days to reach the target dose of 400 mg (100 mg, 200 mg, 400 mg); dosing is continued at 400 mg per day from day 4 through day 14； AZA (75 mg/m^2) is administered subcutaneously on days 1-7, and HHT (1 mg/m^2) on days 1-7.

VEN dose will be reduced by at least 50% in the patients receiving concomitant moderate or strong CYP3A4 inhibitors (eg, azole antifungals). FLT3 inhibitors are allowed in the patients with FLT3 mutations. DLI is administered at day 15 after the initiation of the trial therapy for the relapse patients after allo-HSCT. Once acquiring CRc, the patients are recommended to receive allo-HSCT if donors are available. If donors are unavailable, patients will receive one course of original therapy again and sequential cytarabine-based consolidation therapy. In addition, for the patients undergoing allo-HSCT, sorafenib maintenance post-transplantation was recommended regardless of FLT3 being mutated or not. If patients do not obtain CRc after two courses of the trial therapy, they will proceed to allo-HSCT if donors are available, and patients receive other salvage therapy based on patients' personal preferences after full discussion with physicians if donors are unavailable.

5.3 Follow-up

The clinical data cutoff date is June 30, 2022. Disease assessment including routine blood and BM assessment will be regularly performed post-salvage therapy. For response assessments, bone marrow is evaluated on cycle 1 day 28 and again 1-2 weeks after hematological recovery if the day 28 bone marrow is aplastic. Subsequent bone marrow evaluations are done before and after cycles 2, and then as clinically needed. Adverse events are recorded up to 4 weeks after the discontinuation of the trial therapy, and are graded according to the National Cancer Institute Common Terminology Criteria for Adverse Events version 4.0.

6. Efficacy Assessments

6.1 Definitions

- Relapse is defined as reappearance of leukemic blasts in the peripheral blood or ≥ 5% blasts in the BM as pirate or biopsy not attributable to any other cause or reappearance or new appearance of extramedullary leukemia after achieving CRc.

CR is defined as BM blasts <5%; absence of circulating blasts and blasts with Auer rods; absence of extramedullary disease; ANC $\geq 1.0\times10^9$/L and PLT $\geq 100\times10^9$/L.

CRi is defined as all CR criteria except for incomplete hematologic recovery with neutropenia $<1.0\times10^9$/L and/ or thrombocytopenia $<100\times10^9$/L.

PR is defined as a minimal residual disease of 5-25% with a greater than 50% decrease in leukemic blast percentage.

NR is defined as a failure to obtain CRc or PR.

CRc comprises CR and CRi.

ORR includes CRc and PR.

The MRD level of 0.01% is used as a threshold to distinguish MRD positive (MRD^+) from MRD negative (MRD^-).

OS is defined as the time from treatment until death or censored at the last follow-up.

EFS is defined as the time from treatment until documented failure to achieve CRc, relapse after CRc, or death from any cause, whichever occurred first.

DFS is defined as the time from the date of CRc until relapse, death from any cause, or censored at the last follow-up.

6.2 Primary Efficacy Endpoint

- The primary endpoint is CRc rate after 2 cycles of trial treatment.

6.3 Secondary Efficacy Endpoints

- The secondary endpoints are safety, OS, EFS, DFS and relapse.

6.4 Schedule and methods of Efficacy Assessments

Disease assessment including routine blood and BM assessment will be regularly performed post-salvage therapy. For response assessments, bone marrow is evaluated on cycle 1 day 28 and again 1-2 weeks after hematological recovery if the day 28 bone marrow is aplastic. Subsequent bone marrow evaluations are done before and after cycles 2, and then as clinically needed. Adverse events are recorded up to 4

weeks after the discontinuation of the trial therapy, and are graded according to the National Cancer Institute Common Terminology Criteria for Adverse Events version 4.0.

7. Safety Evaluation

Adverse events are recorded up to 4 weeks after the discontinuation of the trial therapy, and are graded according to the National Cancer Institute Common Terminology Criteria for Adverse Events version 4.0. The study adjudication committee (consisting of experts in hematology, infection, pathology, pharmacy, and statistics) judge whether adverse events are treatment-related or non-treatment-related. Serious adverse events are those that resulted in death, disability, or incapacity, are life-threatening or judged an important medical event, or required hospitalization or prolongation of existing hospitalization.

7.1 Medical History

Each subject's medical history must be obtained at screening. Information on any prior or existing medical conditions will be recorded on the appropriate CRF.

7.2 Vital Signs and Physical Examination

Vital signs and results of physical examination must be documented before enrollment, once a week for the first two months after enrollment. The next 8 items must be performed:

- Physical examination
- Heart rate
- Blood pressure
- Body temperature
- Rate of respiration
- Body weight
- ECOG performance status
- Signs of infection

7.3 Clinical Symptoms

During the study, the patients' clinical symptoms must be documented. The clinical symptoms may be associated with the administration of VEN, or HHT, or AZA reported previously, including rash, dermatitis, erythema, flushing, pruritus, dry skin, alopecia, stomatitis, diarrhea, nausea, vomiting, pancreatitis, weight loss, anorexia, fatigue, fever, headache, sensory neuropathy, myalgia, arthralgia, abdominal pain, cardiac ischaemia, dyspnea, infections, bleeding and hypertension.

7.4 Clinical Laboratory Evaluations

Before initiation of the study, the monitors will document the normal range of each test in every involved laboratory. During the study, the next items must be performed:

- Routine blood: white cell counts, neutrophil cell counts, hemoglobin, and platelet counts
- Hepatic function: total bilirubin (both direct bilirubin and indirect bilirubin must be documented when the total bilirubin elevates), ALT, AST, lactic dehydrogenase, alkaline phosphatase, albumin and total protein
- Renal function: serum creatinine, urea nitrogen and uric acid
- Other biochemical indicators: amylase and lipase
- Electrolytes: sodium, potassium, calcium and magnesium
- Coagulation function: prothrombin time, prothrombin time-international normalized ratio, activated partial thromboplastin time and fibrinogen
- Urinalysis: protein, glucose and erythrocyte
- Electrocardiogram
- Chest imaging examination

8 Adverse Events and Serious Adverse Events (SAEs)

The investigator is responsible for detecting, documenting and reporting events that meet the definition of AE or SAE.

8.1. Definitions

8.1.1 Adverse Events

AE is any untoward medical occurrence in a subject of a clinical investigation, which does not necessarily have a causal relationship to the medicinal product. Therefore, an AE can be any unfavorable and unintended sign, including an abnormal laboratory finding, symptom, or disease (new or exacerbated), whether or not it is considered to be related to the product. This definition includes any newly occurring event or previous condition that has increased in severity or frequency since the administration of the product. However, relapse or death due to relapse should not be recorded as AEs.

8.1.2 Serious Adverse Events

A serious AE is any untoward medical occurrence that, at any dose:

- Results in death
- Is life-threatening
- Requires hospitalization or prolongation of existing hospitalization - ie, the AE requires at least a 24-hour inpatient hospitalization or prolongs a hospitalization beyond the expected length of stay.

Hospitalization or prolongation of existing hospitalization for social reasons will not be reported as SAE.

- Results in disability/incapacity
- Congenital anomaly/birth defect
- Important medical event

Medical or scientific judgment should be exercised in deciding whether SAE reporting is appropriate in other situations. An important medical event is an event that may not result in death, be life-threatening, or require hospitalization, but is clearly of major clinical significance. The AE may jeopardize the subject or require intervention to prevent a serious outcome.

8.2 Assessment of Severity

All AEs are graded according to CTCAE version 4.0.

8.3 Assessment of Causality

The investigator must determine the relationship of each AE and SAE to study treatment. Relationship of AE or SAE to study treatment will be defined according to the following criteria:

- Definite: There is a clear temporal relationship to study treatment, with no other possible cause.
- Possible: A temporal relationship to study treatment is not clear, and alternative etiologies are possible.
- Not related: There is no temporal relationship to study treatment, and/or there is evidence of an alternative cause such as a concurrent medication or illness.

8.4 Recording and Reporting AEs and SAEs

All AEs and SAEs must be recorded in the appropriate CRF, whether or not they are associated to be causally related to study treatment. Each SAE must be reported promptly on the Serious Adverse Event Report Form, and submitted to the Independent Ethics Committee within 24 hours by the investigator. The information recorded on the Serious Adverse Event Report Form will include at least the following: subject number, identity of the event, study drug name and dose, investigator's assessment of the event's severity and relationship to study treatment, and investigator's name and signature. Clinical monitors must collect and verify detailed information of AEs and SAEs when examining original medical records. All AEs and SAEs should be followed up until resolved.

9. Rules of Withdrawal

9.1. Subjects Withdraw from the Study

Subjects can withdraw from the study at any time for any reason without impact on the investigator’s right to treat disease for subjects. Based upon the interest of subjects, the investigator has the right to request subjects to withdraw from the study for any

reason including concomitant disease, AEs or treatment failure. The core group of clinical study reserves the right to request subjects to withdraw from the study for deviation(s) from the protocol, administrative reasons, or other effective or ethical reasons.

The last assessment for subjects must be performed and documented in the CRF regardless of the time and reason for withdrawal. The reason for withdrawal from study participation must be documented in the CRF. All documents related to subjects should be completed. Despite withdrawal from the study, those subjects should be followed up and documented about their diseases until withdrawal of informed consents.

For subjects who withdraw from the study due to concomitant diseases or AEs, the details must be documented in the CRF with other appropriate and valuable data attached.

9.2. Premature Termination of the Study

Reasons for premature termination of the study include external events, repetition of SAEs, growing incidence of treatment-related death and slow enrolment in the study. All subjects will be informed of premature termination of the study by written consents. Any subjects who decide to discontinue participating in the study must report to the principal investigator.

10. Rules of Follow-Up

10.1 Follow-up Period

Starting from randomization.

10.2 Visit Scheduling

Every week for the first three months after enrollment, and then every month until the study is completed.

10. 3 Contents

The contents of every follow-up visit include complaints of subjects, vital signs, physical examination, clinical symptoms and clinical laboratory evaluations (hematology, serum chemistry, urinalysis, electrocardiogram, chest imaging examination, and BM assessment). All of the results must be documented in the original medical record.

11. Data Analysis and Statistical Considerations

11. 1 Sample Size Assumptions

The sample size calculation for the trial was based on the assumption that the VAH regimen would achieve a higher CRc rate compared with historical CRc rate of 45% (on the basis of the venetoclax in combination with HMAs study in R/R AML). To identify a 15% absolute improvement in CRc with VAH regimen, a total of 87 patients was required to provide the study with a significance level of 5% and a power of 80%. After adjusting for a 10% dropout, the total planned sample size was 96 patients.

11.2 Primary Efficacy Endpoint

The primary endpoint is CRc after 2 cycles of trial treatment.

11. 3 Secondary Efficacy Endpoints

The secondary endpoints are safety, EFS, DFS, OS, and relapse.

11.4 Data Analysis Considerations

11.4.1 Analysis Population

The primary population will be the ITT population, which is defined as all subjects enrolled to the VAH treatment. This ITT population will be the basis for the analysis of efficacy and safety endpoints in this study.

11.4.2 Analysis Plan

11.4.2.1 Baseline Data

Baseline characteristics will be summarized and described in a frequency list.

11.4.2.2 Analysis of Efficacy

The definition of efficacy endpoints has been detailed in previous section. The time-to-event endpoints including OS, EFS, and DFS are analyzed by the Kaplan-Meier method and compared using the log-rank tests. The corresponding HR and 95% CI are estimated using the Cox proportional hazards model. Cumulative incidences of relapse are calculated by accounting for competing risks, and non-relapse mortality is a competing risk for relapse. The comparison of the cumulative incidence in the presence of a competing risk is done using the Fine and Gray model. All variables in the Cox models were tested for proportional hazards assumption. Factors that were significant at the 0.1 level from the univariable model were included in the multivariable model. All statistics are analyzed in software R version 4.1.0 (R Development Core Team, Vienna, Austria) or Stata version 15.1 (StataCorp 4905 Lakeway Dr College Station, TX77845, USA) or SPSS version 22.0 (SPSS, Chicago, IL). All statistical tests are two-tailed with a significance level of 0.05.

11.4.2.3 Analysis of Safety

AEs are recorded up to 4 weeks after the discontinuation of the trial therapy, and were graded according to the National Cancer Institute CTCAE version 4.0. The study adjudication committee (consisting of experts in hematology, infection, pathology, pharmacy, and statistics) judge whether adverse events are treatment-related or non-treatment-related. sAEs are those that resulted in death, disability, or incapacity, are life-threatening or judged an important medical event, or required hospitalization or prolongation of existing hospitalization.

12. Materials for the Study

All materials will be provided to study sites and investigators are as follows:

- The study protocol
- Informed consent
- CRF

13. Ethical Considerations

13.1 Responsibility of Investigators

The investigators have the responsibility for guarantee of the clinical study's compliance with the protocol, Chinese good clinical practice (GCP) guidelines and applicable laws and regulations.

13.2 Informed Consent Process

Prior to participation in the study, subjects must be informed about objectives, methods, possible benefits, potential risks and possible discomforts of the study by investigators. They also should be informed that participation in the study would be voluntary, they can withdraw from the study at any time, there is no impact on the treatment of the disease whether they take part in the study and their privacy will be protected.

Subjects or their legally acceptable representative should have enough time to read the inform consent and raise queries. Written informed consent must be obtained from each subject, or their legally acceptable representative.

13.3 Good Clinical Practice

This study will be conducted in accordance with the Declaration of Helsinki and Chinese GCP. The study will be conducted only if it is approved by the ethical review committee of the principal study site. The investigators will guarantee that the study will be conducted in accordance with applicable laws and regulations, scientific and ethical principles of the People's Republic of China. If the protocol needs revision during the study, the revised version must be reapproved by the ethical review

committee of the principal study site. If new data related to study treatment are discovered, the informed consent must be revised and the revision must be reapproved by the ethical review committee of the principal study site and subjects.

13.4 Protection of Subjects' Personal Data

Data collected in the study are limited to the efficacy and safety related to study treatment. Data will be collected and used in accordance with applicable laws and regulations.

14. Administrative Requirements

Neither the investigator nor the applicant can revise the protocol without agreement of the opposite side. All revisions of the protocol must be released by the applicant institution. To insure the integrity, accuracy and reliability of the data, relevant results of examination and treatment must be documented in original medical record and CRF. Independent clinical monitoring is performed regularly by a panel of qualified and experienced study investigators composed of hematologists who are blinded as to the treatment assignments.

15. References

[1] Zhang L, Wang J, Li WB, Yao ZQ.Advances in Research of Small-molecule Inhibitors of Bcl-2 family Proteins. World Notes on Antibiotics. 2014,35(05):196-200+242.

[2] Bradbury DA, Zhu YM, Russell NH. Bcl-2 expression in acute myeloblastic leukaemia: relationship with autonomous growth and CD34 antigen expression. Leuk Lymphoma. 1997;24(3-4):221–228.

[3] Lauria F, Raspadori D, Rondelli D, et al. High bcl-2 expression in acute myeloid leukemia cells correlates with CD34 positivity and complete remission rate. Leukemia. 1997;11(12):2075–2078.

[4] Rao J, Wang YY, Zhou YL, Zhang RY.Over expression of bcl-2 resulting in

resistance todaunorubicin in acute myeloid leukemia cells. Acta Universitatis Medicinalis Anhui. 2017; 52(03):369-372.

[5] Dinardo CD, Pratz K, Pullarkat V, et al. Venetoclax combined with decitabine or azacitidine in treatment-naive, elderly patients with acute myeloid leukemia. Blood 2019;133(1):7–18.

[6] Dinardo CD, Pratz KW, Letai A, et al. Safety and preliminary efficacy of venetoclax with decitabine or azacitidine in elderly patients with previously untreated acute myeloid leukaemia : a non-randomised , open-label , phase 1b study. Lancet Oncol 2018;19(2):216–28.

[7] Wei A, Strickland H, Hou JZ, et al. Venetoclax Combined With Low-Dose Cytarabine for Previously Untreated PatientsWith AcuteMyeloid Leukemia: Results From a Phase Ib/II Study. JCO. 2019; 37(15): 18.01600

[8] Dinardo CD, Kadia T, Rausch CR, et al. Clinical experience with the BCL2-inhibitor venetoclax in combination therapy for relapsed and refractory acute myeloid leukemia and related myeloid malignancies. Am J Hematol 2018;93:401–7.

[9] Weng YW, Tsai CH, Lin CC, et al. Cytogenetics and mutations could predict outcome in relapsed and refractory acute myeloid leukemia patients receiving BCL-2 inhibitor venetoclax. Ann Hematol 2020; 99:501–11.

[10] Moujalled DM, Pomilio G, Ghiurau C, et al. Combining BH3-Mimetics to target both BCL-2 and MCL1 has potent activity in pre-clinical models of acute myeloid leukemia. Leukemia 2019;33(4):905–17.

[11] Konopleva M, Pollyea DA, Potluri J, et al. Efficacy and Biological correlates of response in a phase 2 study of venetoclax monotherapy in patients with acute myelogenous leukemia. Cancer Discov 2016;6(10):1106–17.

[12] Carter BZ, Mak PY, Tao W, et al. Mcl-1/CDK9 targeting by AZD5991/AZD4573 overcomes intrinsic and acquired venetoclax resistance in vitro and in vivo in PDX model of AML through modulation of cell death and metabolic functions. Blood 2018;132(Suppl 1). 768–768.

[13] Pan R, Ruvolo V, Mu H, et al. Synthetic Lethality of combined Bcl-2 inhibition and p53 activation in AML: mechanisms and superior Antileukemic efficacy. Cancer Cell 2017;32(6):748–60. e6.
[14] Su Y, Li X, Edwards H, et al. Venetoclax synergistically enhances the Antileukemic activity of imipridone ONC213, a novel imipridone ONC201 analog, in acute myeloid leukemia. Blood 2018;132(Suppl 1). 3936 LP – 3936.
[15] Liu QW, Wei H. Application and advances in research of homoharringtonine in hematological diseases.Oncology Progress. 2015; 13(04):382-385.
[16] Zhu HH, Jiang H, Jiang Q, Jia JS, Qin YZ, Huang XJ. Homoharringtonine, aclarubicin and cytarabine (HAA) regimen as the first course of induction therapy is highly effective for acute myeloid leukemia with t (8;21). Leuk Res. 2016;44:40–44.
[17] Klanova M, Andera L, Brazina J, et al. Targeting of BCL2 Family Proteins with ABT-199 and Homoharringtonine Reveals BCL2- and MCL1-Dependent Subgroups of Diffuse Large B-Cell Lymphoma. Clin Cancer Res. 2016;22(5):1138–1149.
[18] Yu GP, Xu N, Huang F, et al. Combination of Homoharringtonine with Venetoclax and Azacitidine Excerts Better Treatment Response in Relapsed /Refractory Acute Myeloid Leukemia. Blood, 2020; 136(Supplement 1):26-27. DOI:10.1182/blood-2020-138676
